# Dual Action of Sulfated Hyaluronan on Angiogenic Processes in Relation to Vascular Endothelial Growth Factor-A

**DOI:** 10.1038/s41598-019-54211-0

**Published:** 2019-12-02

**Authors:** Linda Koehler, Gloria Ruiz-Gómez, Kanagasabai Balamurugan, Sandra Rother, Joanna Freyse, Stephanie Möller, Matthias Schnabelrauch, Sebastian Köhling, Snezana Djordjevic, Dieter Scharnweber, Jörg Rademann, M. Teresa Pisabarro, Vera Hintze

**Affiliations:** 1 Institute of Materials Science, Max Bergmann Center of Biomaterials, TU Dresden, Budapester Straße 27, 01069 Dresden, Germany; 2 Structural Bioinformatics, BIOTEC TU Dresden, Tatzberg 47-51, 01307 Dresden, Germany; 3 Institute of Pharmacy, Freie Universität Berlin, Königin-Luise-Straße 2+4, 14195 Berlin, Germany; 4 Biomaterials Department, INNOVENT e.V., Prüssingstraße 27 B, 07745 Jena, Germany; 5 Institute of Structural and Molecular Biology, University College London, Gower Street, Darwin Building, WC1E 6BT London, United Kingdom; 6 Department of Cellular and Molecular Medicine, Glycobiology Research and Training Center, University of California, 92093 San Diego, La Jolla, CA USA

**Keywords:** Molecular biophysics, Growth factor signalling, Computational models

## Abstract

Pathological healing characterized by abnormal angiogenesis presents a serious burden to patients’ quality of life requiring innovative treatment strategies. Glycosaminoglycans (GAG) are important regulators of angiogenic processes. This experimental and computational study revealed how sulfated GAG derivatives (sGAG) influence the interplay of vascular endothelial growth factor (VEGF)_165_ and its heparin-binding domain (HBD) with the signaling receptor VEGFR-2 up to atomic detail. There was profound evidence for a HBD-GAG-HBD stacking configuration. Here, the sGAG act as a “molecular glue” leading to recognition modes in which sGAG interact with two VEGF_165_-HBDs. A 3D angiogenesis model demonstrated the dual regulatory role of high-sulfated derivatives on the biological activity of endothelial cells. While GAG alone promote sprouting, they downregulate VEGF_165_-mediated signaling and, thereby, elicit VEGF_165_-independent and -dependent effects. These findings provide novel insights into the modulatory potential of sGAG derivatives on angiogenic processes and point towards their prospective application in treating abnormal angiogenesis.

## Introduction

Sulfated glycosaminoglycans (sGAG) are linear, negatively charged polysaccharides consisting of repetitive disaccharide units (D.U.), which interact with a variety of mediator proteins and, thereby, modulating their biological activity^1,2^. Native GAG differ in their sulfation content and pattern as well as their monosaccharide composition, which results in a high complexity of GAG-mediated biological functions^3–7^.

Vascular endothelial growth factor (VEGF)-A is a 45 kDa homodimeric glycoprotein and the most potent and specific regulator of physiological and pathological angiogenesis^8^. It is required for the chemotaxis and differentiation of endothelial precursor cells (angioblasts), endothelial cell (EC) proliferation, the direct assembly of ECs into vascular structures (vasculogenesis) and angiogenic remodeling^9^. Alternative splicing of VEGF-A gives rise to at least eight isoforms^10,11^. VEGF_165_ is the predominant isoform in human tissues and contains a heparin (Hep) binding domain (HBD)^10^. VEGF_165_ exerts its biological effects through binding to the high affinity receptor tyrosine kinases VEGF receptor-1 (VEGFR-1) and -2 (VEGFR-2), as well as to the co-receptor neuropilin-1 (NRP-1), all of which are predominantly expressed on ECs^10^. However, the major receptor for the mitogenic, angiogenic and vascular permeability enhancing effects of VEGF is VEGFR-2^12^. Upon ligand binding, VEGFR-2 undergoes dimerization and strong autophosphorylation of the cytoplasmic domains on specific tyrosine residues resulting in a mitogenic, chemotactic and prosurvival signal^8,12,13^. GAG such as Hep and heparan sulfate (HS) have profound effects on VEGF_165_ function, not only by binding VEGF_165_ directly, but also by interacting with VEGF receptors and NRP-1^14–16^. *In vitro* studies demonstrated that cell surface HS interaction with VEGF_165_ enhance VEGF_165_-induced phosphorylation of VEGFR-2 and increase mitogenic activity as well as endothelial tube formation^17–20^. In addition, binding of VEGF_165_ to VEGFR-2 was affected by the size, degree of sulfation (DS), sugar ring stereochemistry and conformation of Hep^21–23^.

Currently, in a constantly aging population with increasing number of multimorbid patients^24^, controlling angiogenic factors represents a very important goal for regenerative medicine and tissue engineering in terms of improving healing processes, particularly in injured vascularized tissues such as bone and skin. Innovative biomaterials containing GAG derivatives with defined sulfation degree and pattern are promising tools for selectively influencing their molecular recognition by target mediator proteins such as growth factors and, thereby, modulating their biological activity. In previous studies, a regulatory effect of hyaluronic acid (HA) derivatives on angiogenic processes was revealed. On the one hand sulfated HA (sHA) interfered with the TIMP-3-mediated inhibition of VEGF-A mediated signaling. On the other hand, sHA-containing HA/collagen-based hydrogels were found to directly stimulate the proliferation of a porcine EC line^25^. However, these findings were limited to selected HA derivatives restricting a detailed and comprehensive understanding of the potential dual action of sulfated HA on angiogenic processes. Against this background, in the present study, the interactions between VEGF_165_ or its HBD domain and a broad range of HA and chondroitin sulfate (CS) derivatives with defined sulfation degrees and patterns were analyzed in comparison to native GAG using surface plasmon resonance (SPR) and computer-based molecular modeling techniques. Furthermore, the consequences of these interactions on VEGF_165_/VEGFR-2 complexation and the biological function of VEGF_165_ were evaluated *in silico* up to the atomic detail and in 2D *in vitro* cell culture experiments using human umbilical vein endothelial cells (HUVEC). The impact of different GAG was verified in a 3D *in vitro* angiogenesis assay by analyzing sprouting of HUVECs in the absence and presence of VEGF_165_. This allows for an in-depth understanding of the dual GAG activities and reveals whether it translates into a pro- or anti-angiogenic effect on ECs in a complex system.

## Materials and Methods

### Materials

Hyaluronan (HA) (from Streptococcus, MW = 1.1 × 10^6^ g mol^−1^) was obtained from Aqua Biochem (Dessau, Germany). Sulfur trioxide/dimethylformamide complex (SO_3_–DMF, purum, 97%, active SO_3_ 48%) as well as sulfur trioxide/pyridine complex (SO_3_–pyridine, pract.; 45% SO_3_) were acquired from Fluka Chemie, (Buchs, Switzerland). Hep extracted from porcine intestinal mucosa and the specific VEGFR-2 inhibitor SU1498 were available from Sigma-Aldrich (Schnelldorf, Germany). Hep hexasaccharide (dp 6) was obtained from Iduron (Manchester, UK). Recombinant human VEGF_165_ (293-VE-010/CF) and neutralizing VEGFR-2 antibody (MAB3572-100) were obtained from R&D Systems (Wiesbaden-Nordenstadt, Germany). For SPR measurements, the Series S Sensor Chips C1, CM5 and CM3, the Amine Coupling Kit and HBS-EP (10x) from GE Healthcare Europe GmbH (Freiburg, Germany) were used. The VEGF_165_ HBD was purified as previously described^26^.

### Preparation of polymeric and oligomeric GAG derivatives

**Figure 1:**
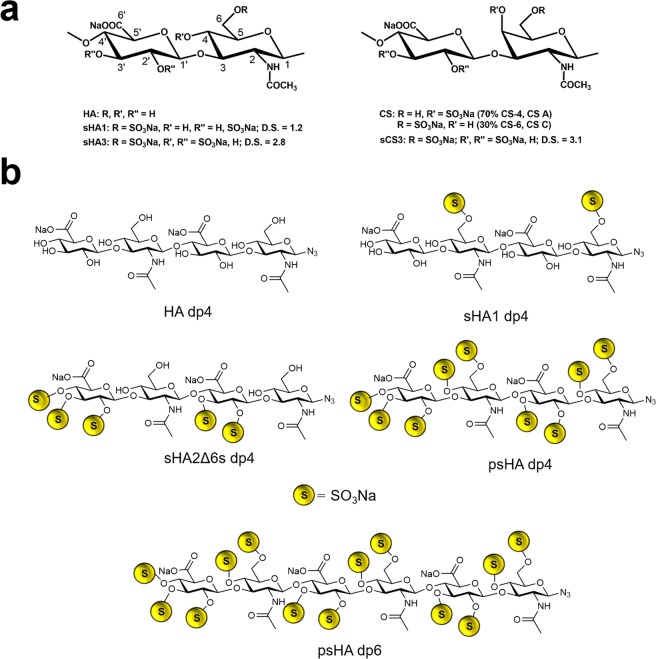
Structural characteristics of polymeric and oligomeric GAG.

The polymeric HA and CS derivatives were synthesized and characterized according to previous protocols^27–29^. Analytical data of the used polymeric GAG derivatives (Fig. 1a) are summarized in Table 1. Preparation and characterization of oligomeric HA derivatives (Fig. 1b) was performed as previously described^30–32^.

**Table 1:** Characteristics of polymeric GAG derivatives.

Sample	HA	sHA1	sHA3	CS	sCS3	Hep
D.S.^a^	—	1.2	2.8	0.8	3.1	2.2
M_w_ [g/mol]^b^	48 300	27 300	28 700	20 700	19 900	18 000
Sulfate group distribution^c^	—	6, 2′, 3′	462′, 463′, 62′3′	4, 6	462′, 463′, 62′3′	2′6, 2N, 6

^a^Degree of sulfation (D.S.) as determined by elemental analysis, ^b^weight-average (Mw) values as revealed with laser light scattering (LLS) detection and ^c^the sulfate group distribution as detected by nuclear magnetic resonance.

### Surface plasmon resonance analyses

#### Immobilization of VEGF_165,_ HBD of VEGF_165_ and VEGFR-2 to sensor chip surfaces

For SPR analysis, a BIACORE T100 instrument (GE Healthcare) was used. VEGF_165_ was immobilized to Series S Sensor Chip CM3 at 25 °C in HBS-EP running buffer (0.01 M HEPES, (pH 7.4), 0.15 M NaCl, 3 mM EDTA, 0.055 surfactant P20) using the amine coupling reaction according to the manufacturer’s protocol (GE Healthcare). 5–10 µg/ml growth factors diluted in sodium acetate buffer, pH 5.5 were injected at 5 µl/min until an immobilization level of approximately 1200 RU was achieved^33^ A surface without immobilizing one of the growth factors was used as a reference. The HBD of VEGF_165_ (VEGF_165_-HBD) was covalently bound to a Series S Sensor Chip CM5 at 25 °C in HBS-EP by amine coupling. Therefore, 30 µg/ml HBD dissolved in sodium acetate puffer (pH 5.5) was streamed for 45 s over an activated sensor chip surface (flow: 10 µl/min) resulting in an immobilization level of about 900 RU. 50 µg/ml VEGFR-2 dissolved in sodium acetate buffer (pH 4.5) was immobilized to a Series S Sensor Chip C1 at 5 µl/min to an immobilization level of approximately 220 RU.

#### SPR analysis of GAG binding to immobilized growth factors

Interaction studies were performed as described previously^33^. Briefly, GAG (diluted in HBS-EP) were injected for 300 s at 30 µl/min (at 37 °C in HBS-EP) and binding levels were recorded 10 s before injection stop. The injection was followed by a 10 min dissociation phase in running buffer at a flow rate of 30 µl/min. The sensor chip surface was regenerated after each sample injection with 5 M NaCl in 40 mM NaOH or 5 M NaCl in 10 mM NaOH in case of the VEGF_165_ HBD for 60 s at a flow rate of 30 µl/min. The baseline was allowed to stabilize for 1000 s with running buffer prior to injection of the next sample. Binding parameters were evaluated using the BIACORE T100 evaluation software 2.03. Data were double referenced by the response of the reference surface and the response of HBS-EP buffer alone relative to a baseline report point. Furthermore, binding levels were corrected for the respective molecular weight of the GAG derivatives to consider the fact that the SPR binding response is related to a mass increase at the sensor chip surface.

### Isothermal titration calorimetry

Titrations of 60 µM psHA dp4 and 50 µM psHA dp6 to 15 µM VEGF_165_-HBD were carried out using a Microcal PEAQ-ITC calorimeter (Microcal, Malvern Panalytical, Malvern, GB) at 25 °C. VEGF_165_-HBD was dialyzed against 8 mM phosphate buffer (8 mM NaH_2_PO_4_∙2H_2_O, 2 mM KCl, pH 7.5) without DTT with 10 wt-% DMSO to reduce noise before measurements. To evaluate the influence of the salt concentration on binding of sulfated oligohyaluronans to VEGF_165_-HBD, additional measurements were performed in phosphate buffered saline, PBS (10 mM NaH_2_PO_4_∙2H_2_O, 2 mM KH_2_PO_4_, 3 mM KCl, 140 mM NaCl, pH 7.4) without DTT with 10 wt-% DMSO. The synthesized oligosaccharides psHA dp4 and psHA dp6 were dissolved in the same buffer to ensure a reasonable baseline. For each titration 19 to 25 injections of 1.5 and 1 µl of titrant, respectively, were conducted at 180 s intervals, while stirring at 750 rpm. Three different control experiments were performed: buffer in buffer titration, a 60 µM solution of psHA dp4 titrated to buffer and buffer titrated to a 15 µM VEGF_165_-HBD solution. Raw data were integrated, normalized and the titration curve fitted using Microcal PEAQ-ITC analysis software provided by Microcal. Binding curves were fitted to the single site model.

### Molecular modeling and molecular dynamics simulation

#### Modeling of VEGF_165_ dimer and VEGF_165_/VEGFR-2 ternary complex

**Figure 2:**
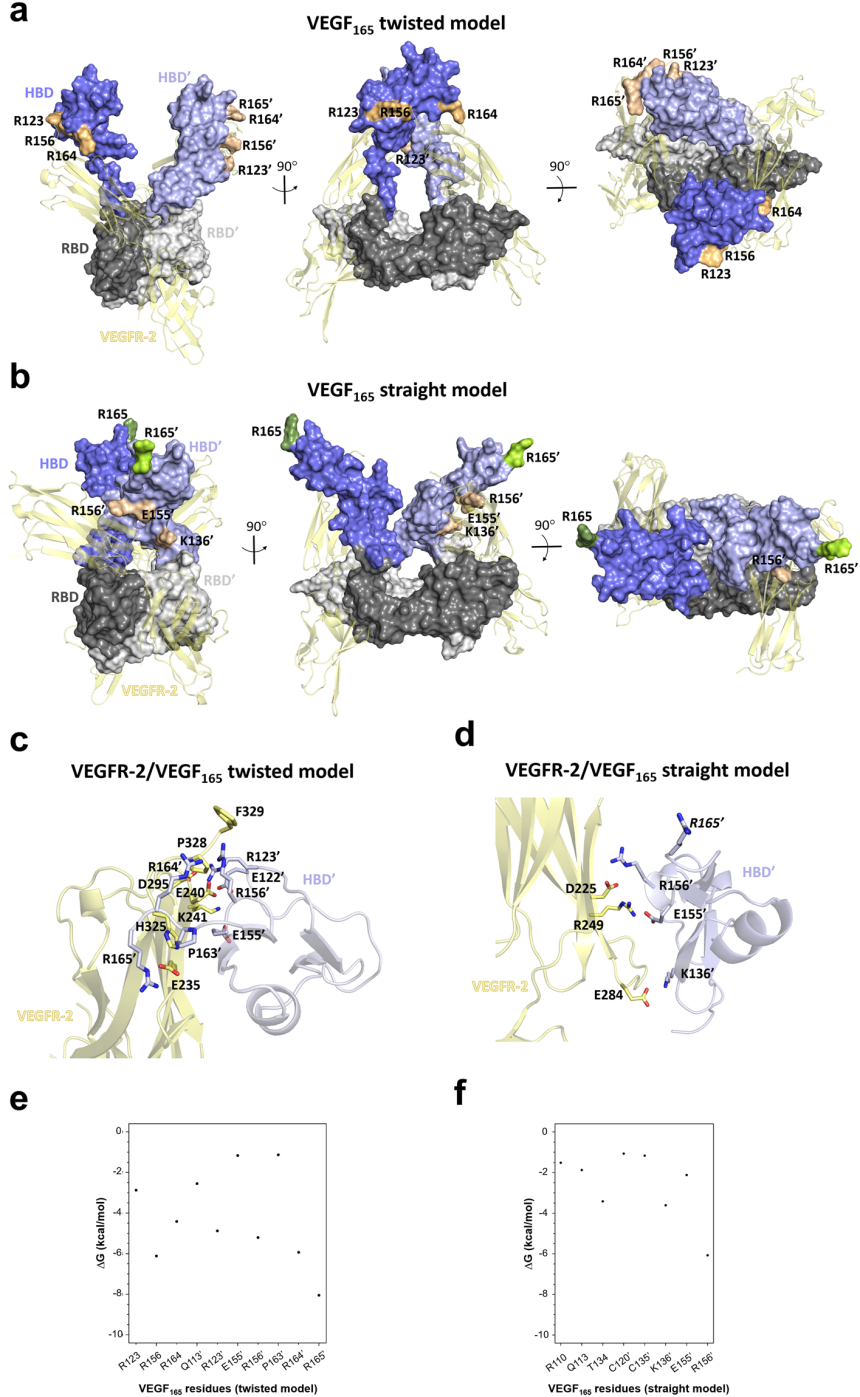
Molecular modeling of the 3D structure of VEGF_165_ dimer and energy contribution analysis. Two distinct conformations of the VEGF_165_ dimer in complex with VEGFR-2 refined by MD simulations are obtained: “*twisted*” (**a**) and “*straight*” (**b**). VEGF_165_ models (*twisted* and *straight*) and VEGFR-2 are depicted in molecular surface and cartoon representation, respectively. The RBD is shown in gray (dark and light representing each monomer), the HBD in blue (dark and light representing each monomer) and VEGFR-2 in yellow. Charged residues at the HBD of VEGF_165_ interacting with VEGFR-2 are highlighted in pale (dark and light for each monomer) and numbered (VEGF_165_ monomers are distinguished by a comma). Residue R165, which is essential for NRP-1 recognition, is not interacting in the “*straight*” model and is shown in green (dark and light for each monomer). Close-up of the MD refined complex of VEGFR-2 with VEGF_165_-HBD *twisted* (**c**) and *straight* (**d**) models with relevant interacting residues in sticks, colored by atom type and labeled. The no interacting residue R165 in the VEGF_165_ *straight* model is labeled in italic. Per-residue energy analysis (calculated with MM-GBSA from MD simulations) of most contributing residues of VEGF_165_-HBD *twisted* (**e**) and *straight* (**f**) models in binding to VEGFR-2.

The full three-dimensional (3D) VEGF_165_ dimer structure was built taking into account the following experimentally available 3D crystal structures from the Brookhaven protein data bank (PDB) as templates: (i) the high-resolution crystal structures of the N-terminal region of VEGF corresponding to its receptor binding domain (VEGF-RBD), which is a dimer structure (PDB ID 2VPF, 1.9 Å)^34^, (ii) the NMR structure of the C-terminal region of VEGF corresponding to its heparin binding domain (VEGF-HBD; PDB ID 1VGH)^35^, and (iii) the structure of the complex of VEGFR-2 with the VEGF receptor binding domain (RBD) (complex 1:1, PDB ID 3V2A, 3.2 Å)^36^. Unfortunately, the full structure of VEGF_165_ dimer as such is not experimentally available. The amino acid Arg110 acting as a link between the N- and C- terminal domains is not resolved in any of the available experimentally solved structures. Therefore, in order to model the 3D spatial disposition of VEGF’s N- and C-terminal domains (i.e. RBD and HBD, respectively) with respect to each other in the dimer form of VEGF and with respect to the VEGFR-2, we did the following. First of all, based on literature about the stoichiometry of the VEFG-VEGFR-2 signaling complex from which it is known that the dimer VEGF signals through complexing with 2 receptor molecules^13^, we generated the complete structure of the dimer VEGF-RBD/VEGFR-2 complex (1:2) by applying the corresponding symmetry operations in PDB ID 3V2A. In the resulting structure, we modeled the missing residue Arg110 in extended conformation at the C-term on one of the VEGF-RBD (RBD-Arg110). We connected RBD-Arg110 to HBD-Ala111 in each of the 20 structures comprised in the HBD NMR ensemble (PDB ID 1VGH), and we examined the resulting configurations of the HBD domain with respect to the two receptor molecules and the two RBD by taking into consideration available information on the potential role of VEGF_165_-HBDs in the regulation of VEGFR-2 phosphorylation suggesting that they could also interact with the receptor^17^. This analysis allowed us to be able to exclude those configurations of HBD presenting steric clashes either with the receptor or with the RBD molecules. The best resulting model fulfilling the above-mentioned requirements resulted from the structure number 4 from the HBD NMR ensemble. As a next step, we performed a Arg110-Ala111 ϕ and φ dihedral scan and looked for interfacial electrostatics and shape compatibility of HBD with the receptor molecules and avoiding any steric hindrances. This resulted in two representative models of VEGF_165_ monomer consisting of two well-differentiated orientations of HBD with respect to VEGFR-2: i) HBD interacting with the close by VEGFR-2 and ii) HBD interacting with the farther VEGFR-2 (Fig. S1). In order to finally build the VEGF_165_ dimer complexed with two receptor molecules, we applied a twofold symmetry operation to the previously modeled VEGF_165_ monomer based on the symmetry exhibited by the VEGF-RBD/VEGFR-2 crystallographic structure (PDB ID 3V2A). The intermolecular disulfide bonds between the VEGF_165_-RBD monomers were correspondingly modeled. Next, and because receptor residues Ser264 to Lys271 and Lys278 to Gly282 are not resolved in the experimentally available X-ray VEGFR-2 structure, in order to complete the full complex structure those missing residues were modeled taking as template the VEGFR-2 structure in complex with VEGF-C (PDB ID 2X1W, 2.7 Å)^37^. As a last step, the two VEGF models were energetically refined in complex with the two receptor molecules by applying molecular dynamics (MD). Modeller as implemented in Discovery Studio (Accelrys Software) was used for the molecular modeling^38^, and AMBER14 was used for the MD simulations (40 ns; for details see MD simulations section)^39^. After refinement of the two models, it was observed that the HBD were disposing themselves with respect to the receptor molecules in two distinguishable arrangements: one, which we named “*twisted*” in which the RBD of one VEGF_165_ monomer and the HBD of the other VEGF_165_ monomer interact with the same VEGFR-2 molecule, and another one, which we named “*straight*” allowing the interaction of the HBD and RBD of a VEGF_165_ monomer with the same VEGFR-2 molecule (see Fig. 2 for details). For each model (*twisted* and *straight*), the lowest energy structure was selected for further docking studies with the GAG derivatives.

#### Modeling of GAG derivatives

The following GAG derivatives were modeled in AMBER14^39^ and MOE^40^ as previously described^32,41^: Hyaluronan (HA), sulfated hyaluronan (sHA1, sulfated either at position C4 or C6 of the disaccharide unit), high-sulfated hyaluronan (sHA3, sulfated at positions C4, C6 and C3′ in each disaccharide unit), chondroitin sulfate (CS, sulfated either at position C4 or C6 of the disaccharide unit), high-sulfated chondroitin sulfate (sCS3, sulfated at positions C4, C6 and C3′ in each disaccharide unit), tetrameric (dp4) hyaluronan azide derivatives HA, sHA1, sHA2Δ6s, psHA and psHA dp6 (Fig. 1). Based on previous work^42^, the hexamer GAG length (dp6) was considered as representative of polymeric GAG for our docking studies.

#### Molecular docking

Computer-based binding was performed by docking calculations^32,41^ of the GAG derivatives with each of the VEGF_165_ dimer refined models and with the respective receptor VEGFR-2. For this, we used Autodock 3^43^. Autogrid3 was used to calculate the atomic potential of each structure covering the full surface with a grid box and spacing grid of 126 Å × 126 Å × 126 Å and 0.710 Å for VEGF_165_ and 126 Å × 126 Å × 126 Å and 0.580 Å for VEGFR-2. The GAG molecules were treated completely flexible, and the previously refined protein structures were considered rigid. The Lamarckian genetic algorithm with an initial population size of 300 and a termination condition of 10000 generations and 9995 × 10^5^ energy evaluations was used. A total of 1000 independent runs were carried out. Spatial clustering of the top 50 docking solutions was performed with the DBSCAN algorithm^44^ as previously described^45^. From each of the clusters obtained, a representative GAG pose was selected for further refinement of the corresponding GAG-protein complex.

#### Molecular dynamics simulations

The GAG-protein complexes selected as representative from the docking studies were further refined by MD simulations in AMBER14^39^ as previously described^32,41^. Charges were taken from the GLYCAM 06-j force field^46^ for the different sulfated hyaluronan units and from the literature for sulfate groups^47^. AMM1-BCC charges were used for the azide group^48^. Parameters for the GAG part were taken from the GLYCAM-06j force field^46^, and for the proteins from the ff14SB force field^39^. Missing parameters of the azide group were taken from the General Amber Force Field (GAFF)^49^. Each GAG-protein complex was solvated in a truncated octahedral box of TIP3P water molecules and neutralized with Na^+^ or Cl^−^ counterions. MD simulations were preceded by two energy-minimization steps: (i) only the solvent and ions were relaxed with position restraints for the solute (500 kcal/mol·Å^2^) using 1000 steps of steepest descendent minimization followed by 500 steps of conjugate gradient minimization; (ii) the entire system was minimized without restraints applying 3000 cycles of steepest descendent and 3000 steps of conjugate gradient equilibration. Then the system was heated up from 200 K to 300 K in 20 ps with weak position restraints (10 kcal/mol·Å^2^). Langevin temperature coupling with a collision frequency γ = 1 ps^−1^ was used at this step. The system was equilibrated under constant pressure of 1 atm using periodic boundary conditions (NPT conditions) at 300 K for 50 ps. A total of 20 ns MD simulation was carried out at 300 K NPT conditions for each complex. The SHAKE algorithm was used to constrain all bonds involving hydrogen atoms. A time step of 2 fs was used during SHAKE algorithm. A cutoff of 8 Å was applied to treat the non-bonded interactions, and the Particle Mesh Ewald (PME) method was used to treat long-range electrostatic interactions. MD trajectories were recorded every 10 ps. The pyranose rings in the GAG molecules were harmonically restrained. In the case of the complexes between VEGF_165_ and the oligohyaluronan derivatives, a 40 ns MD simulation was carried out. Trajectories were visualized with VMD^50^ and evaluated in terms of intermolecular H-bonds by using the CPPTRAJ module implemented in AMBER. At least 10% of hydrogen bond occupancy was taken as criterion for dynamic hydrogen bond formation. Energy decomposition per residue as well as binding free energy post-processing analysis of the last 300 frames from the MD simulations were performed in implicit solvent using the MM-GBSA method^51,52^ as implemented in AMBER14. Data analysis was carried out with the R-package^53^. Figures were created with PyMOL^54^.

### Cell Culture

#### Cultivation of HUVECs

HUVECs (VEGF pre-screened; PromoCell, Heidelberg, Germany) were maintained in EC medium (PromoCell Endothelial Basal Medium, EBM) supplemented with 2% (v/v) fetal calf serum (FCS), 1% penicillin/streptomycin (Pen/Strep), 5 ng/ml rhEGF, 10 ng/ml rhbFGF, 20 ng/ml R3 IGF-1, 0.5 ng/ml rhVEGF, 1 µg/ml ascorbic acid, 22.5 µg/ml heparin and 0.2 µg/ml hydrocortisone at 37 °C in a humidified atmosphere with 5% CO_2_. Cells were grown to 80% confluence before splitting or stimulation. Passages of HUVECs used for experiments were not higher than four.

#### Phosphorylation of VEGFR-2

Phosphorylation of VEGFR-2 was determined as described previously^33^. HUVECs were seeded in 6-well plates at a density of 10,000 cells/cm^2^ in complete EC medium for 24 h at 37 °C. Prior to stimulation HUVECs were serum-starved for 24 h in EBM containing 0.1% BSA, 0.2% FCS and 1% Pen/Strep. Cells were treated for 10 min with 2.6 nM VEGF_165_ and 200 µM D.U. GAG, pre-incubated for 10 min at 37 °C. Activation of VEGFR-2 in HUVEC cells stimulated with VEGF_165_/GAG complexes was determined using two sandwich ELISA kits (Biotechne) detecting phosphorylated VEGFR-2 (P-VEGFR-2) and total VEGFR-2, respectively, according to the manufacturer’s protocol. As a control, VEGFR-2 phosphorylation was blocked by 100 nM inhibitor SU1498.

#### 3D *in vitro* Angiogenesis Assay

The protocol used was adapted from PromoCell (Heidelberg, Germany) and Korff *et al*. (Korff T *et al*., Exp Cell Res 297:415–23, 2004). The modified angiogenesis assay was performed as described in^33^. HUVECs were trypsinized and resuspended in EBM containing 2% FCS and 10% (v/v) methocel (Sigma-Aldrich) stock solution. 1 × 10^3^ cells/well were seeded in 96-well round-bottom well plates to generate spheroids within 18–24 h at 37 °C, 5% CO_2_. Spheroids were harvested (5 min, 1000 × g, RT) and embedded in gels of 3 mg/ml collagen type I and 1x M199 cell culture medium (Sigma-Aldrich), adjusted to a neutral pH using 0.2 M NaOH. Here, 600 µl of the pH-adjusted collagen I-M199 solution were added to 100 spheroids in methocel-FCS medium (1:1) and mixed carefully. 400 µl/well of the methocel-collagen solution containing the spheroids was added to 48-well plate and incubated at 37 °C for 30 min. Each well contained 50 spheroids, which were stimulated afterwards according to the respective experiment using EBM containing 0.5% FCS, 1% P/S, 25 ng/ml VEGF and 100 µM D.U. GAG. Growth factor and GAG were incubated for 10 min at 37 °C to form complexes and then added to the samples. For neutralization experiments 10 µg/ml anti-VEGFR-2 antibody (VEGFR-2 Ab) were used. Spheroids were treated with 100 µl stimulating solution per well for 24 h. All components (growth factor, GAG, neutralizing antibody) were added as 5x stock of the final concentration according to the final volume of 500 µl in the wells. 10 spheroids per condition were randomly selected and evaluated regarding the individual sprout length, the calculation of the cumulative sprout length and the number of sprouts analyzed using ImageJ.

### Statistical analysis

All experiments were performed at least in triplicate, and results are presented as means ± standard deviation (SD). One-way ANOVA or two-way ANOVA with Tukey or Bonferroni post-test, respectively, were applied. P values < 0.05 were considered statistically significant.

## Results

### Molecular models of VEGF_165_ and VEGF_165_/VEGFR-2

The complete structure of the VEGF_165_ dimer has not been yet experimentally resolved. The structures of the VEGF_165_-RBD at the N-terminus and its VEGF_165_-HBD at the C-terminus are available separately in the Protein Data Bank (PDB), but no full VEGF_165_ structure is available containing both domains connected. In order to build the VEGF_165_ dimer structure, we have first made use of relevant biological information referred to the fact that the signaling complex requires the interaction of one VEGF_165_ dimer with two VEGFR-2 molecules (see Methods section for details) and, furthermore, that the VEGF_165_-HBDs could be implicated in the regulation of VEGFR-2 phosphorylation^17^ and, therefore, they could also interact with the receptor. Thus, the experimentally not resolved connecting residue Arg110 was modeled by attaching the HBD and the RBD of one monomer crystallographic structure taking into account the criteria of avoiding steric clashes (see section 2.5.1 for details). After performing a Phi and Psi dihedral angle scan on the linked residues Arg110-Ala111, two representative and distinguishable orientations with respect to the VEGFR-2 molecules were selected. The application of a twofold symmetry operation as observed in the RBD structure to the resulting VEGF_165_ monomer structure along the y-axis (see Fig. S1) led two VEGF_165_ dimer models that were further MD refined in complex with two VEGFR-2 molecules (see section 2.5.1 for details). Each VEGF_165_ dimer conformation was named according to the orientation of the HBDs and the VEGFR-2 molecules. The first one consists of a “*twisted*” conformation, in which the RBD of one VEGF_165_ monomer and the HBD of the second monomer interact with the same VEGFR-2 monomer molecule. The second one shows a “*straight*” conformation, which allows the interaction of the HBD and RBD of the same VEGF_165_ monomer with the same VEGFR-2 monomer molecule (Fig. 2).

Binding of the VEGF_165_ dimer to two VEGFR-2 molecules was more favorable for the *twisted* than for the *straight* model (ΔG_VEGF165*twisted*/VEGFR-2_ = −217.6 ± 16.3, ΔG_VEGF165*straight*/VEGFR-2_ = −149.7 ± 9.7). In the *twisted* model, residues Arg123, Arg156, Arg164 and Arg165 belonging to the HBD domains are involved in the recognition of VEGFR-2, whereas in the case of the *straight* model, those correspond to residues Lys136, Glu155 and Arg156. Thus, Arg156 constitutes a common recognition residue in both models (Fig. 2). Both VEGF_165_ conformations could potentially co-exist in their receptor-free state. However, taking into account previous studies on the interaction of VEGF with other molecules such as the NRP-1 co-receptors^10,55^, the VEGF_165_ *straight* model would be the most biologically relevant one as it would allow simultaneous interactions with VEGF receptors and co-receptors (Fig. 2).

### SPR analysis of GAG binding to VEGF_165_ and molecular recognition of GAG by VEGF_165_

**Figure 3:**
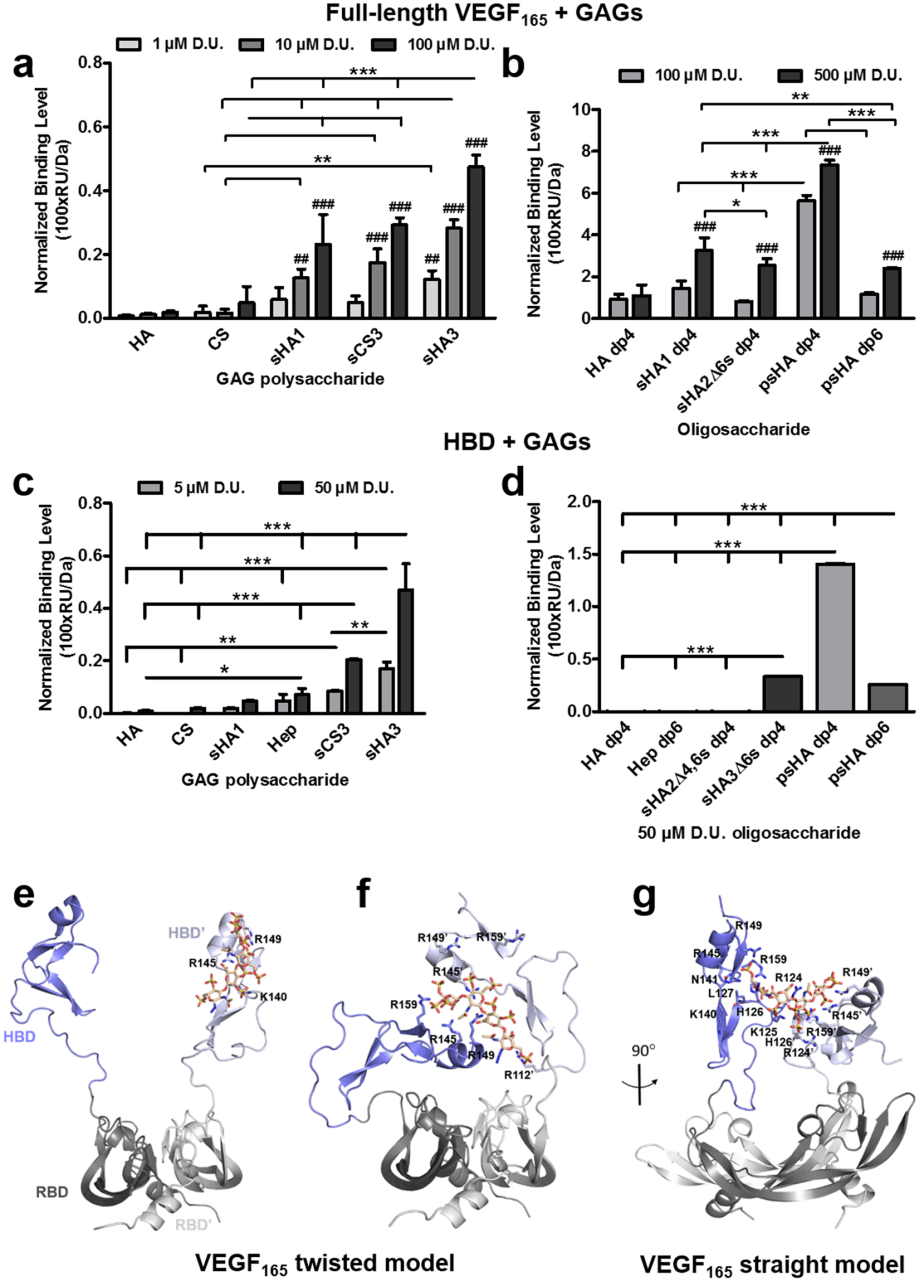
Interaction of immobilized VEGF_165_ and HBD with solute GAG derivatives as determined by SPR. Binding levels for the interaction of (**a**) polymeric and (**b**) oligomeric GAG to VEGF_165_ and HBD (**c**,**d**) are displayed [adapted and modified from^33^]. All values represent the mean ± SD of n = 3 and are given as relative to baseline response and corrected for the respective molecular weight of GAG derivatives. Two-way ANOVA: *p < 0.05; **p < 0.01; ***p < 0.001 vs. respective treatment; ^##^p < 0.01; ^###^p < 0.001 vs. (**a**) HA or (**b**) HA dp4. Predicted recognition of psHA dp4 by VEGF_165_ dimer. (**e**) Initial complex VEGF_165_ *twisted*/psHA dp4. (**f**) Refined VEGF_165_ *twisted*/psHA dp4 (40 ns). (**g**) Refined VEGF_165_ s*traight*/psHA dp4 (40 ns). VEGF_165_ is depicted in cartoon style, and relevant interacting residues are shown in sticks and colored by atom type. The RBD is shown in gray (dark and light representing each monomer), and the HBD is shown in blue (dark and light representing each monomer). psHA dp4 is shown in stick representation and colored by atom type.

The interaction of solute GAG derivatives with immobilized VEGF_165_ was analyzed by SPR. While HA showed only a weak interaction with VEGF_165_, a higher sulfation and concentration of the polymeric GAG derivatives led to stronger binding, with sHA3 showing the highest binding strength (Fig. 3a). The ranking of binding strength for the polymeric GAG was as follows: HA ≤ CS < sHA1 < sCS3 < sHA3. Interestingly, sHA derivatives revealed higher binding compared to CS derivatives with a different sugar backbone but a comparable DS. The ranking of binding strength for the oligomeric derivatives to VEGF_165_ revealed the following: HA dp4 < sHA2Δ6s dp4, psHA dp6 < sHA1 dp4 < psHA dp4 (Fig. 3b), highlighting the importance of sulfation at the C6 position of the *N*-acetylglucosamine (GlcNAc) unit as well as the polymerization degree. Additional SPR measurements (Fig. 3c,d) using the VEGF_165_-HBD clearly demonstrated the direct binding of GAG derivatives to the HBD and support the GAG rankings for full-length VEGF_165_. It is of note that, while polymeric Hep bound to HBD, no binding could be detected for Hep dp6.

Molecular docking was used to predict GAG recognition by the VEGF_165_ dimer. The resulting GAG-protein complexes were energy refined by MD. For VEGF_165_, the *twisted* and *straight* models resulting from the MD refinement in the presence of VEGFR-2 were used. For both VEGF_165_ conformations, all studied GAG were predicted to bind to the VEGF_165_-HBD, which is further supported by experimental studies using the HBD and GAG derivatives (Fig. 3c,d). In the case of the *twisted* model, GAG were predicted to bind in two different modes: parallel and/or perpendicular orientation with respect to the HBDs. For the *straight* model, two distinct binding modes were observed depending on the GAG type. A parallel binding mode to HBD was predicted for HA (both, polymeric and oligomer), sHA1 and sHA2Δ6s. In case of all sulfated polymeric GAG derivatives and persulfated oligohyaluronan derivatives, a parallel-curved binding mode was observed in which the GAG was bridging the two HBDs (Fig. S2a–e). This could be feasible considering that the separation between both HBDs is in the same range as the GAG dp6 lengths (*ca*. 30 Å).

Independently of the protein conformation, we observed that all studied sulfated polymers initially bound to one VEGF_165_-HBD were acting as a kind of “*molecular glue*” originating molecular recognition modes in which the GAG molecule sticks on the two VEGF_165_-HBDs along the MD energy refinement. This HBD-GAG-HBD stacking configuration appeared around the first 10 ns and was preserved through the rest of the MD simulation. For most of the tetrameric oligohyaluronan derivatives, a comparable stacking configuration was observed at about 20 ns. Therefore, to further explore and fully refine the obtained stacking configuration, for the oligohyaluronan derivatives the MD simulation was elongated to a total of 40 ns, in which such configuration remained stable (Fig. S3). In the case of the non-sulfated HAs the stacking configuration was not observed.

The binding energies computed with MM-GBSA^51,52^ for the polymeric GAG indicated that binding to VEGF_165_ was, in general, more favorable for the *straight* model. Noticeably, independently of the VEGF_165_ model used for our calculations, the binding obtained increased with the DS of the GAG (Table 2), which is in agreement with our experimental observations (Fig. 3a). In the case of the VEGF_165_ *twisted* model, binding was more favorable for HA than for CS derivatives of the same DS. Moreover, binding was more favorable for sHA1 with GlcNAc sulfated at C6 position than for the other investigated monosulfated GAG derivatives, and similar than sCS3 (Table 2, Fig. S2f). In contrast, in the *straight* model the predicted binding was similar for mono- as well as high-sulfated HA and CS. The binding energies obtained for the oligohyaluronans in the VEGF_165_ *twisted* model resulted in the following ranking: HA dp4 < sHA2Δ6s dp4 < sHA1 dp4 < psHA dp4 < psHA dp6 (Table 2) and, as observed for the polymeric analogues, GlcNAc of sHA1 showed more favorable binding energies than those from sHA2Δ6s (Fig. S2g). Interestingly, the binding energy ranking obtained for the *straight* model agreed well with the binding data obtained from the SPR experiments. The psHA dp4 showed the most favorable binding energy to the VEGF_165_ *straight* model among all oligohyaluronan azides (Table 2).

**Table 2:** MM-GBSA binding free energies obtained for GAG in complex with VEGF_165_^a^.

GAG	ΔG_GAG-VEGF165*twisted*_ (kcal/mol)	ΔG_GAG-VEGF165*straight*_ (kcal/mol)
HA dp4	−24.9 ± 3.3	−16.7 ± 3.4
HA dp6	−13.4 ± 6.1	−38.8 ± 5.8
sHA1 dp4	−61.5 ± 6.3	−55.5 ± 5.8
sHA1 dp6 (sulfated at C4)	−35.8 ± 4.5	−59.2 ± 7.4
sHA1 dp6 (sulfated at C6)	−66.8 ± 8.3	−55.3 ± 9.5
sHA2Δ6s dp4	−50.0 ± 10.6	−60.7 ± 10.3
sHA3 dp6 (sulfated at C4,C6,C3′)	−72.9 ± 12.7	−106.7 ± 10.8
CS4 dp6 (sulfated at C4)	−34.0 ± 4.8	−65.2 ± 8.8
CS6 dp6 (sulfated at C6)	−46.4 ± 7.3	−52.6 ± 8.0
sCS3 dp6 (sulfated at C4,C6,C3′)	−67.6 ± 9.9	−108.6 ± 9.5
psHA dp4	−82.0 ± 9.0	−153.7 ± 9.5
psHA dp6	−111.3 ± 7.6	−121.9 ± 11.3

^a^Shown for the energetically most favored binding modes.

The detailed analysis of the interactions established in the refined VEGF_165_ *straight* model indicated that the HBD-GAG-HBD stacking complex was stabilized by favorable contacts between psHA dp4 and residues Arg123, Arg124, Lys125, His126, Leu127, Lys140, Arg145, Arg149 and Arg159 of both HBDs, and Asn141 for one of the HBDs. Furthermore, the MD-based per-residue energy calculations showed a major number of favorable interacting residues of VEGF_165_ with psHA dp4 in comparison to psHA dp6. In particular, interactions of Arg124, His126, Leu127 and Arg159 with psHA dp4 were more favorable than with psHA dp6, while psHA dp6 led to interactions that were more favorable with Lys140 and, in lesser extent, with Lys125 in comparison to the tetramer analogue (Fig. S4). In the *twisted* model, the HBD-GAG-HBD stacking structure obtained for psHA dp4 exhibited a lower number of interactions, which involved some of the residues observed in the *straight* model (Arg145, Arg149 and Arg159 for both VEGF_165_ HBDs, and Arg110, Ala111 and Arg112 for one HBD) (Fig. 3f,g). The differences in binding energies obtained for psHA dp6 and dp4 with VEGF_165_ twisted model were due to the more favorable interactions of Lys140, Lys147, Lys162 and Arg164 with the hexamer analogue in comparison to the tetramer derivative, in which the major contributors to the binding energies were Arg110, Ala111, Arg112 and Arg145 (Fig. S4). In general, independently of the VEGF_165_ model, residues Arg145, Arg149 and Arg159 appear to be crucial and common for the recognition of all investigated sGAG (Fig. S4). Interestingly, previous VEGF_165_ mutagenesis studies indicated that simultaneous mutations involving Arg123, Arg124, Lys140, Arg145, Arg156 and Arg159 could almost abolish Hep binding to the protein^56,57^, which supports our molecular models. Likewise, the *straight* model was the one matching maximum number of significant mutagenesis interactions (Fig. S4). In conclusion, our findings suggest that GAG recognition by VEGF_165_ occurs through a HBD-GAG-HBD stacking complex involving both HBDs. Although in the absence of receptor the *twisted* and *straight* conformations of VEGF_165_ may be possible and both support a stacking conformation when recognizing a GAG molecule, in the case of the signaling complex (VEGF_165_ dimer and the VEGFR-2 dimer), and based on our models and the experimental indications on NRP-1 recognition^10,55–57^ as well as previous mutagenesis studies, the *straight* conformation appears to be the one being functionally relevant.

Isothermal titration calorimetry (ITC) was performed to validate the stoichiometry of the predicted HBD-GAG-HBD stacking conformation. The titration of a psHA dp4 and psHA dp6 to VEGF_165_-HBD in independent measurements (Figs. S5, S6 and S7) indicated a mean binding stoichiometry of N = 0.503 and N = 0.545 respectively, corresponding to one oligohyaluronan binding two VEGF_165_-HBDs. Binding of the psHA dp4 was observed to be driven by a negative binding enthalpy of −5 kJ mol^-1^ and by a strong positive entropic contribution of +32 kJ mol^-1^ (for TΔS) resulting in the dissociation constant *K*_*D*_ of 364 ± 48 nM (Table 3, Fig. S6). The mean binding stoichiometry and the dissociation constant of sulfated oligohyaluronans binding to VEGF_165_-HBD were unaffected by the salt content of the buffer (ITC titrations done in phosphate buffer: N = 0.493, *K*_*D*_ = 378 ± 21 nM, Fig. S5; ITC titrations done in PBS buffer: N = 0.503, *K*_*D*_ = 364 ± 48 nM, Fig. S6). The higher sulfated, oligomeric hexahyaluronan psHA dp6 revealed three times stronger binding compared to psHA dp4 (psHA dp4: *K*_*D*_ = 364 ± 48 nM, psHA dp6, *K*_*D*_ = 124 ± 29 nM, Fig. S7) in agreement with the predicted ranking from our MM-GBSA calculations for the proposed VEGF_165_ *twisted* model (Table 2). The differences in binding between the tetra- and hexahyaluronan derivatives obtained through SPR and ITC could be explained considering the fact that ITC experiments were performed in solution, allowing the simultaneous complex formation of two HBD molecules with one GAG, which may be the reason for higher affinity for dp6 compared to dp4. In contrast, SPR experiments were performed with an immobilized HBD, which does not allow the formation of equivalent complexes that are possible in the ITC experiments. Furthermore, our theoretical models predicted a more favorable binding energy for psHA dp6 when considering the VEGF_165_ *twisted* model in comparison to the straight (Table 2). It should be considered that in our theoretical models two HBDs can simultaneously interact with the GAG, but they have some conformational restriction because of their covalent attachment to the RBDs (in ITC the HBD have more conformational freedom due to the fact that they do not have any kind of immobilization). The observed differences in binding could simply be a consequence of the accessibility of the HBD in different conditions.

**Figure 4:**
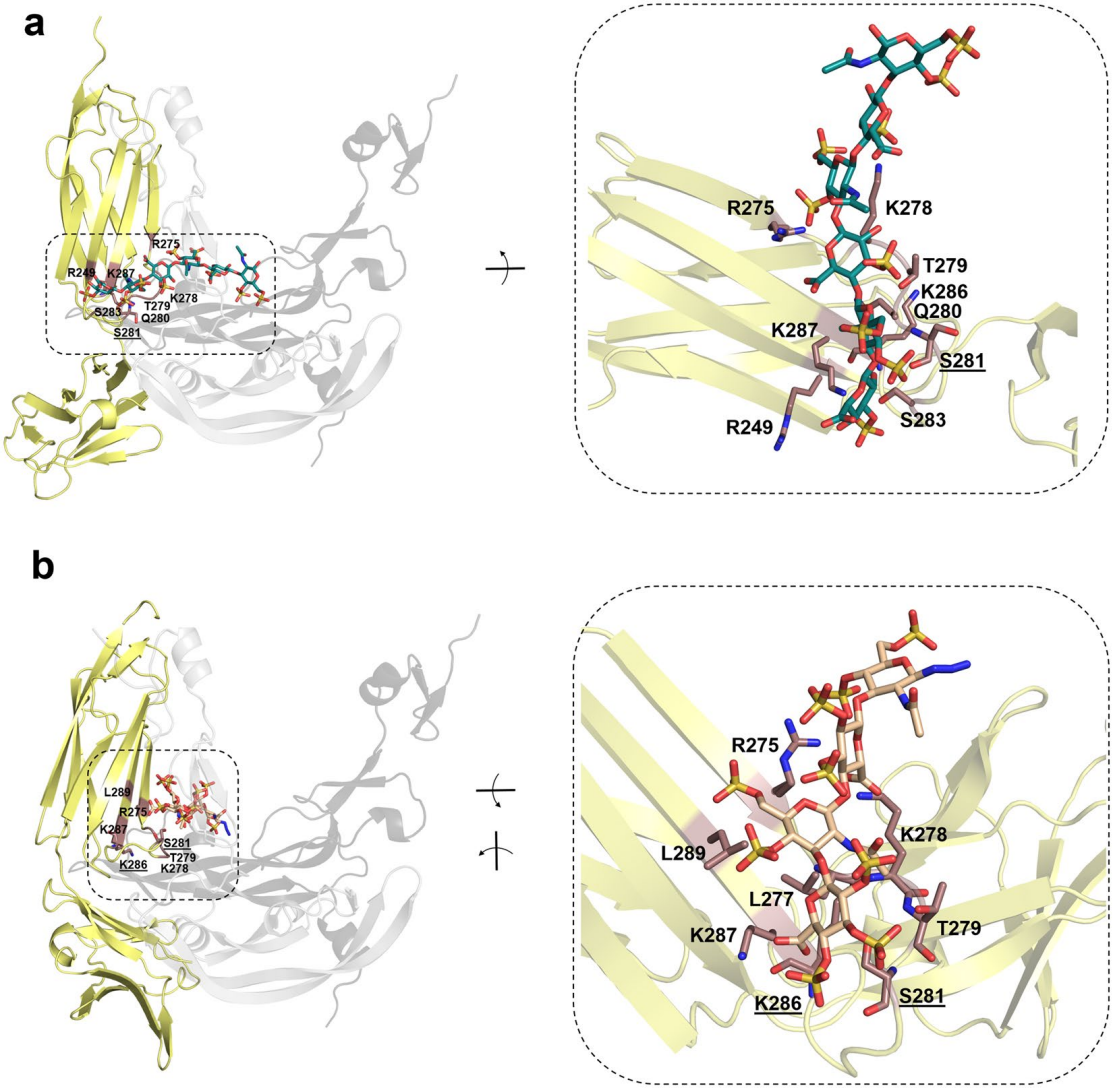
Molecular modeling of the interaction of VEGFR-2 with sGAG derivatives. Representative snapshots from 20 ns MD simulations of most favorable binding sites and modes of (**a**) sHA3 dp6 (ΔG_VEGFR-2/sHA3_ = −22.8 ± 3.2 kcal/mol) and (**b**) psHA dp4 (ΔG_VEGFR-2/psHA dp4_ = −43.0 ± 4.4 kcal/mol) in complex with VEGFR-2. VEGFR-2 is depicted in yellow cartoon representation with relevant interacting residues colored brown and labeled. Receptor residues recognizing GAG and also VEGF_165_ are shown in sticks colored by atom type and are labeled (underlined). For illustrative purposes, VEGF_165_ (not taken into account for calculations) is shown in grey cartoon transparency (dark and light representing each monomer) in the left panels. GAG derivatives are shown in stick and colored by atom type.

To investigate the effect of GAG binding to VEGF_165_ on the recognition of its receptor VEGFR-2, the VEGF_165_/VEGFR-2 interface was analyzed and compared to the GAG/VEGF_165_ recognition sites (Figs. 2 and S4). In case of the VEGF_165_ *twisted* model, residue Arg164, which is involved in binding of sGAG, is also engaged in VEGFR-2 recognition. In contrast, in the VEGF_165_ *straight* model GAG and VEGFR-2 do not compete for the same recognition site. Nevertheless, taking into account that sGAG promote HBD-GAG-HBD stacking complex formation with VEGF_165_, it could be assumed that the binding strength of the HBD toward VEGFR-2 would decrease with the consequent weakening of VEGF_165_/VEGFR-2 complex formation. To shed light onto this possible mechanism, the recognition of sHA3 dp6 by the ternary complex (*i.e*. two VEGFR-2 receptors and a dimeric VEGF_165_ molecule) was further investigated computationally. A HBD-GAG-HBD stacking configuration between VEGF_165_/sHA3 was also obtained in the presence of the two receptors. Indeed, for both conformations, *twisted* and *straight*, sHA3 stabilized the complex (ΔG_VEGF165*twisted*-sHA3/VEGFR-2_ = −252.6 ± 11.5, ΔG_VEGF165*straight*-sHA3/VEGFR-2_ = −172.1 ± 11.1), which resembles previous experimental observations with Hep in a length- and concentration-dependent-manner^21,22^. Interestingly, some of the interactions between the receptors and the VEGF_165_-HBDs were lost upon sHA3 binding and formation of the HBD-GAG-HBD stacking configuration (Fig. S8). In particular, Arg156 of VEGF_165_ constitutes a common missing interactive residue in both VEGF_165_ conformations, while, Asp63 and Glu64 in the VEGF_165_-RBD increase their interactions with VEGFR-2. The recognition of GAG by VEGFR-2 was also investigated (Fig. 4, Table S1). Here, high-sulfated HA derivatives did not show strong binding toward the receptor but, nevertheless, they established specific interactions with Ser281 and Lys286, which were also involved in the recognition of VEGF_165_.

**Table 3:** Results of the ITC measurements for VEGF_165_-HBD and psHA dp4.

compound	c^a^_protein_	c^a^_ligand_	N^b^	ΔG^c^	ΔH^d^	TΔS^e^	K_D_^f^	$\bar{{\bf{x}}}$ (K_D_)^g^
psHA dp4^**h**^	15	60	0.505	−36.8	−17.3	+19.5	357 ± 21	378 ± 21
			0.481	−36.5	−17.1	+19.4	398 ± 10	
psHA dp4^**i**^	15	60	0.523	−36.4	−3.7	+32.7	431 ± 245	364 ± 48
			0.557	−37.0	−7.2	+29.7	341 ± 73	
			0.429	−37.1	−4.6	+32.5	320 ± 200	
psHA dp6^**i**^	15	50	0.628	−39.0	−4.8	+34.2	149 ± 80	124 ± 29
			0.409	−40.5	−5.7	+34.8	83 ± 30	
			0.599	−39.2	−5.0	+34.2	140 ± 65	

^a^Concentration in µM, ^b^molar binding ratio of the ligand-protein interaction (stoichiometry), ^c^binding free energy in kJ∙mol^−1^, ^d^binding enthalpy in kJ∙mol^−1^, ^d^entropic contribution term in kJ∙mol^−1^, ^f^dissociation constant in nM, ^g^mean of the calculated *K*_*D*_ values, ^h^ITC measurements were performed in 8 mM phosphate buffer with 2 mM NaCl, ^i^ITC measurement were performed in 10 mM PBS buffer with 140 mM NaCl.

### Phosphorylation of VEGFR-2 in the presence of GAG

**Figure 5:**
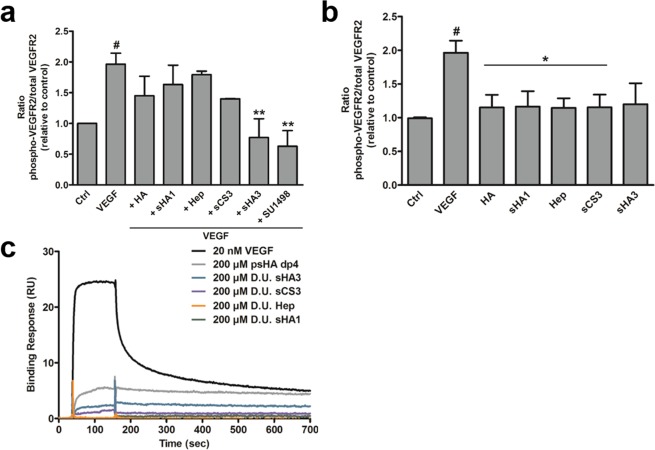
Influence of GAG derivatives on VEGF_165_-mediated phosphorylation of VEGFR-2 (**a**,**b**) and direct interaction of solute GAG derivatives with immobilized VEGFR-2 (**c**). (**a**) HUVEC cells were stimulated with GAG for 10 min. Cells were lysed afterwards and lysates were applied to a specific phospho- and a total VEGFR-2 sandwich ELISA. The amount of phospho-VEGFR-2 relative to total VEGFR-2 is shown. Values represent the mean ± SD of n = 2. One-way ANOVA: ^#^p < 0.05 vs. Ctrl; *p < 0.05 vs. VEGF_165_. (**b**) HUVEC cells were stimulated with VEGF_165_ alone or pre-formed VEGF/GAG complexes for 10 min. Cell lysates were applied to a specific phospho- and a total VEGFR-2 sandwich ELISA. The amount of phospho-VEGFR-2 relative to total VEGFR-2 is shown. SU1498, a specific VEGFR-2 inhibitor was used as a reference for reduced phosphorylation. Values represent the mean ± SD of n = 3. One-way ANOVA: ^#^p < 0.05 vs Ctrl; **p < 0.01 vs. VEGF_165_ alone. (**c**) One representative sensorgram out of three independent measurements is shown [adapted and modified from^33^].

In order to determine the direct effect of solute GAG on VEGFR-2 activation, the receptor phosphorylation in the absence or presence of VEGF_165_ was studied via ELISA. The presence of HA, sHA1, sCS3 and Hep had no significant influence on VEGF_165_-mediated VEGFR-2 phosphorylation in comparison to VEGF_165_ alone (Fig. 5a). In contrast, sHA3 displayed a significant inhibitory effect on VEGFR-2 phosphorylation comparable to the VEGFR-2 inhibitor SU1498. In the presence of sCS3, VEGFR-2 phosphorylation was reduced as well compared to VEGF_165_ alone, but not significantly and, therefore, not comparable to SU1498. In the absence of VEGF_165_, no effect of GAG alone on VEGFR-2 phosphorylation was observed (Fig. 5b). Nevertheless, SPR binding data indicated a weak interaction between sGAG and VEGFR-2 (Fig. 5c).

### Influence of GAG derivatives on VEGF_165_-mediated sprouting of HUVEC spheroids

**Figure 6:**
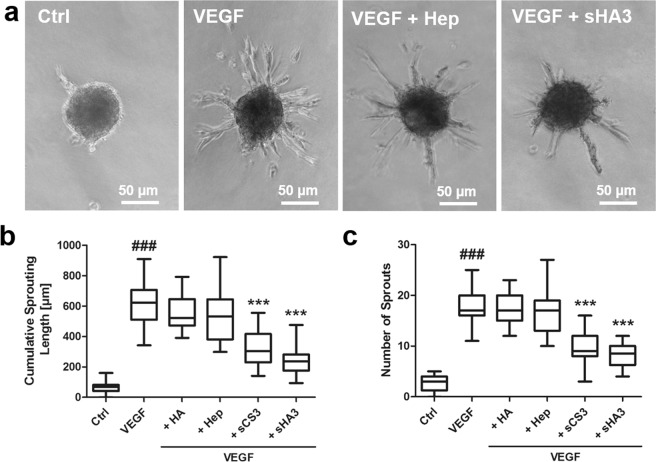
Biological consequences of VEGF_165_/GAG interaction on VEGF_165_-mediated sprouting of HUVEC spheroids. 1 × 10^3^ cells were seeded in medium containing 10% methyl cellulose to form spheroids, which were embedded in methyl cellulose/collagen-I gels and treated with VEGF_165_ alone or pre-formed VEGF_165_/GAG complexes. (**a**) Sprouting of HUVEC spheroids determined by light microscopy. (**b**) Cumulative sprouting length calculated using ImageJ and (**c**) number of sprouts. For (**a**) one representative microscope picture is shown and for (**b**) and (**c**) values represent the mean ± SD of n = 3 with 10 spheroids evaluated for each independent experiment. One-way ANOVA***p < 0.001 vs. VEGF_165_ alone; ^###^p < 0.001 vs. Ctrl. [adapted and modified from^33^].

The biological consequences of VEGF_165_/GAG interaction were further evaluated in a 3D *in vitro* angiogenesis assay. HUVEC spheroids were treated with growth factor alone or pre-formed growth factor/GAG complexes. Treatment with VEGF_165_ led to the formation of numerous sprouts compared to the untreated control (Fig. 6). In the presence of HA and Hep no change in sprouting behavior of spheroids compared to VEGF_165_ treatment was determined. In contrast, sCS3 and sHA3 significantly inhibited VEGF_165_-mediated spheroid sprouting displayed by a reduced cumulative sprouting length and a reduced number of sprouts (Fig. 6b,c).

### Growth factor-independent effects of GAG on spheroid sprouting

**Figure 7:**
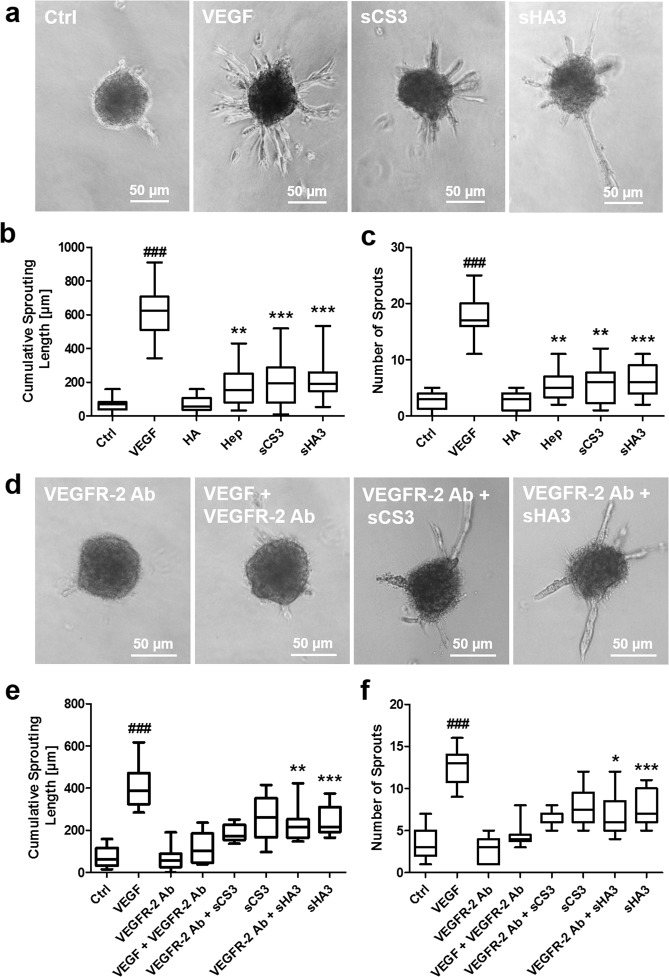
Impact of GAG derivatives and role of VEGFR-2 for the pro-angiogenic effect of GAG derivatives on sprouting of HUVEC spheroids. VEGF_165_ was used as a positive control for spheroid sprouting in a 3D *in vitro* angiogenesis assay. (**a**) Sprouting of HUVEC spheroids determined by light microscopy. (**b**) Cumulative sprouting length calculated using ImageJ and (**c**) number of sprouts. For (**a**) one representative microscope picture is shown and for (**b**) and (**c**) values represent the mean ± SD of n = 3. One-way ANOVA: **p < 0.01; ***p < 0.001 vs. Ctrl. (d-f) Cells were treated with a VEGFR-2 neutralizing antibody (VEGFR-2 Ab) and GAG. (**d**) Sprouting of HUVEC spheroids determined by light microscopy. (**e**) Cumulative sprouting length calculated using ImageJ and (**f**) number of sprouts. For (**d**) one representative microscope picture is shown and for (**e**) and (**f**) values represent the mean ± SD of n = 3 with 10 spheroids evaluated for each independent experiment. One-way ANOVA: *p < 0.05; **p < 0.01; ***p < 0.001 vs. VEGF + VEGFR-2 Ab; ^###^p < 0.001 vs. Ctrl. [adapted and modified from^33^].

Treatment of HUVEC spheroids with GAG in the 3D *in vitro* angiogenesis assay revealed no induction of sprouting for HA itself (Fig. 7). In contrast, sGAG stimulated sprouting in a sulfation-dependent manner. Cumulative sprouting length and sprout number were comparably increased by sCS3 and sHA3 (Fig. 7b,c) indicating that these GAG were exhibiting VEGF-independent effects. To evaluate whether VEGFR-2 recognition by sGAG (Figs. 4 and 5c) initiates the pro-angiogenic effect of GAG observed in the 3D *in vitro* angiogenesis assays, additional experiments with VEGFR-2-blocking antibody were performed. VEGF-dependent sprouting of HUVEC spheroids was suppressed in the presence of 10 µg/ml anti-VEGFR-2 (Fig. 7d–f). However, the anti-VEGFR-2 antibody was unable to abrogate the effect of sCS3 and sHA3 on HUVEC spheroids, and these experiments revealed a comparable sprouting intensity as observed for GAG alone (Fig. 7e,f). For the neutralizing VEGFR-2 Ab used in the present study, no interaction with sHA3 and sCS3 was observed in SPR binding studies.

## Discussion

Chemically defined sHA derivatives are promising to functionalize biomaterials since their sulfate groups modulate binding and biological activity of growth factors, like VEGF_165_ thereby influencing healing processes^30,42,58,59^. Pro-angiogenic and anti-angiogenic effects of GAG were previously described regarding their impact on VEGF biological activity, although often restricted to heparin^17,19,60–64^, which is known for its heterogeneity regarding the carbohydrate backbone, sulfation degree and pattern^1^.

Thus, in the present study, the potential dual action of sGAG was investigated with a broad range of HA and chondroitin sulfate (CS) derivatives with defined sulfation degrees and patterns in comparison to native GAG. First, the interaction between sGAG and VEGF_165_ or its HBD domain was analyzed using SPR and molecular modeling techniques. Second, the consequences of these interactions on VEGF_165_/VEGFR-2 complexation and the biological function of VEGF_165_ were evaluated *in silico* up to the atomic detail and in 2D *in vitro* cell culture experiments using HUVECs. Finally, the impact of different GAG was verified in a 3D *in vitro* angiogenesis assay by analyzing sprouting of HUVECs in the absence and presence of VEGF_165_. GAG displayed a concentration- and sulfation-dependent interaction with full-length VEGF_165_ and with its HBD. Interestingly, a preferred binding of sHA compared to CS derivatives with a comparable DS was observed, suggesting that the carbohydrate backbone of the GAG significantly influences the interaction. This is in line with previous findings for TGF-β1 and BMP-2^27,65^. The reason for this might be that the molecular geometries in the carbohydrate backbones of sCS (GalNAc) and sHA (GlcNAc) render the respective sulfated groups to interact differently. Furthermore, the differences in the sulfation extent of specific positions within the D.U. might be another explanation. While sHA derivatives were completely sulfated at C6 of GlcNAc, and to a lesser extent at C2′ and C3′ of GlcA and C4 of GlcNAc, native CS contained 70% CS-4, sulfated at C4 of GalNAc before additional complete sulfation at C6 of GalNAc leading to sCS3^28^.

The establishment of two distinct VEGF_165_ dimer molecular models, *straight* and *twisted*, which could co-exist in solution allowed us to investigate the mechanisms for GAG-VEGF_165_ recognition at atomic detail. In line with SPR, GAG were predicted to bind to the HBD at the C-terminus of VEGF_165_. In addition, SPR and free energy calculations demonstrated that GAG binding to HBD occurs in a sulfation-dependent manner. However, when comparing sulfated HA and CS derivatives, the differences in binding obtained from the theoretical models were not as apparent as with SPR analysis. For most of the analyzed GAG, the binding energy obtained was more favorable for the *straight* conformation. Our studies pointed toward a HBD-GAG-HBD stacking complex structure with the GAG embedded between the two HBD domains of the VEGF_165_ dimer. Furthermore, our theoretical models revealed that VEGF_165_ residues Arg145, Arg149 and Arg159 are crucial for sGAG recognition, which agree with previous mutagenesis studies^56,57^. Moreover, our analysis indicated that the VEGF_165_ *straight* model exhibited maximum number of significant GAG interactions matching the mutagenesis data (Fig. S4) shedding light on its biological relevance. In line with these findings, the fact that the VEGF_165_ *straight* model allows simultaneous interactions with VEGF receptors and co-receptors^55^ additionally supports its functional relevance.

In order to gain experimental evidence on the predicted molecular recognition of sGAG by VEGF_165_, the binding of psHA dp4 and dp6 to VEGF_165_-HBD was assessed by ITC. Calorimetric investigation revealed a mean stoichiometry close to that of a ternary complex suggesting that one psHA dp4/dp6 molecule binds to two VEGF_165_-HBD, which validates the predicted HBD-GAG-HBD stacking recognition mode. In these experiments, the physiological salt concentration did not affect the stoichiometry and *K*_*D*_ values obtained for psHA dp4. Formation of the ternary complex was primarily driven by a positive entropic contribution. These thermodynamic data suggest that binding of two VEGF_165_-HBD by the sGAG is impelled - at least in part - by the desolvation of hydrophobic protein surfaces that are exposed to solvent in the GAG-free protein dimer and are joined together by sGAG binding. Interestingly, the formation of ternary complexes of psHA dp4 with other proteins such as IL-8 and IL-10 was driven by a strongly negative enthalpic contribution with a small negative entropic effect instead^32^.

SPR interaction analysis revealed that a tetrasaccharide is a sufficient length for sHA to interact with VEGF_165_, and a sulfation-dependent binding, as observed for polymeric sHA, was confirmed. Furthermore, the C6 sulfation of GlcNAc was shown to play an important role, as the binding strength for the higher sulfated sHA2Δ6s dp4, non-sulfated at C6, was lower compared to C6-sulfated sHA1. Our calculations supported this for the VEGF_165_ *twisted* model, while small differences in binding energies were obtained for the *straight* model. Interestingly, a higher dp does not necessarily enhance binding strength, as binding of psHA dp4 to full-length VEGF_165_ and VEGF_165_-HBD was stronger compared to dp6. This suggests that tetrasaccharides could bind to additional regions of VEGF_165_, as previously described for TIMP-3^66^.

ITC results revealed a stronger binding of psHA dp6 to VEGF_165_-HBD than psHA dp4 (Table 3, Figs. S5, S6 and S7), which is in line with the theoretical binding energies obtained through the VEGF_165_ *twisted* model. The discrepancy between SPR and ITC psHA dp4 and dp6 binding data could be understood by considering the different experimental conditions in which the corresponding experiments are performed in each case. VEGF_165_-HBD is immobilized in SPR experiments, which may preclude certain complex formation accessible in solution phase in the ITC experiments. Our theoretical models which consider VEGF_165_ dimer showed how two HBDs can simultaneously interact with one GAG. Furthermore, the covalent attachment of the HBDs to the RBDs makes their conformational freedom lower than in the case of the free VEGF_165_-HBD in solution (ITC).

Considering that VEGF is assumed to act as a single entity, it can form a continuous binding surface for the interaction with large GAG^67^. Hence, the two identical HBDs of a dimer can be occupied by the same GAG chain. Robinson *et al*. showed that Hep dp7 is sufficient to fully occupy the Hep binding cleft of VEGF^68^. However, in case of smaller GAG it can be assumed that the two sites of the dimer could interact with the GAG independently of each other. Therefore, more than one molecule of psHA dp4 might interact with the VEGF_165_ dimer. In contrast to Hep oligosaccharides, psHA dp6 might be sufficient to stick on both HBDs, which in our models appear separated from each other by a distance equivalent to that of the GAG length. Our theoretical models suggested, nevertheless, a different molecular mechanism for the recognition of short sHA oligosaccharides. In the case of the VEGF_165_ *twisted* model, psHA dp4 and dp6 were initially located along one HBD or the two HBDs, while the recognition of the VEGF_165_ *straight* model by such HA derivatives resulted initially in a simultaneous binding to both VEGF-HBDs. Interestingly, after MD refinement, both models offered an equilibrated HBD-GAG-HBD stacking structure; mechanism which was indeed substantiated by ITC measurements as stated above. Our calculations showed more favorable interactions for psHA dp4 than dp6 toward the *straight* model (Fig. S4f, Table 2), which could explain the strikingly strong binding of psHA dp4 among the oligohyaluronans found by SPR. Interestingly, as stated above, this VEGF_165_ *straight* model is supported by previous studies to be the most biologically relevant form because it allows simultaneous interactions with VEGF receptors and co-receptors^55^. On the other hand, our predictions indicated more favorable binding for psHA dp6 than dp4 toward the VEGF_165_ *twisted* model, which is in line with the obtained *K*_*D*_ data through ITC experiments. Differences in GAG accessibility to the HBDs may be responsible of the distinct binding obtained in different conditions. In conclusion, the binding strength to VEGF_165_ depends on the specific structural features of the oligosaccharides including their DS, sugar ring stereochemistry and conformation.

The interaction of VEGF_165_ with sHA3 led to a decreased VEGFR-2 mediated biological activity displayed by an impaired VEGFR-2 phosphorylation and a reduced HUVEC spheroid sprouting. However, EC sprouting was not completely blocked. First evidence regarding the inhibitory effect of sHA3 on the pro-angiogenic activity of VEGF_165_ was given by Rother *et al*.^25^. However, experiments were carried out only in a 2D model using a porcine EC line. Furthermore, there has been no comparison to other chemically and native, sulfated GAG. In the present study, a trend for reduced phosphorylation was observed also in the presence of sCS3 together with a significantly reduced sprouting of EC spheroids. Both high-sulfated GAG, therefore, exerted anti-angiogenic effects regarding the activity of VEGF_165_. This could be explained by previous findings^30^ revealing an impaired interaction of VEGF with VEGFR-2 in the presence of sGAG. Likewise, our atomic detailed models revealed that sGAG and VEGFR-2 were competing for Arg164 at the binding surface of VEGF_165_ in the *twisted* model. Furthermore, such molecular recognition mechanism would impair further interactions with other co-receptors such as NRP-1, which could negatively influence signaling. According to our predictions, and regardless of the possible coexistence of distinguishable *twisted* and *straight* conformations, the recognition of sGAG by VEGF_165_ points toward the formation of a HBD-GAG-HBD stacking complex structure in which the VEGF_165_-HBDs would lose contacts toward VEGFR-2, especially with Arg156 (Fig. S8), and consequently weakening the strength for HBD-receptor binding. This molecular mechanism could offer a plausible explanation to the experimental results, which is in accordance with previous observations showing the potential regulatory role of VEGF_165_-HBDs on VEGFR-2 phosphorylation^17^.

Still, sHA1 did not lead to a reduced phosphorylation of VEGFR-2, which is in accordance with a modest inhibitory effect on VEGF/VEGFR-2 interaction as previously shown^30^. It is in agreement with its weaker binding to full-length VEGF_165_ and VEGF_165_-HBD revealed by SPR and computer-based analysis. Here, free VEGF_165_ might still be able to interact with the receptor leading to an activation and subsequent signal transduction. Also for Hep, no significant effect on receptor activation and HUVEC spheroid sprouting was observed, even though a pronounced blocking effect on VEGFR-2 binding was reported^30^. These opposing results could be explained taking into account that in our cell culture experiments Hep might also interact with other proteins present, in contrast to SPR experiments where VEGF_165_ and VEGFR-2 are the only possible GAG interaction partners. Beneficial effects for Hep on several growth factor/receptor interactions are described, but differences between Hep sources can lead to high structural variability, resulting in different outcomes of experiments^17,21,22^.

In summary, high-sulfated GAG derivatives impair the biological activity of VEGF_165_ by hindering receptor activation and subsequent downstream signaling, which might be due to an impaired receptor binding. These findings suggest a potential local application of these particular GAG derivatives as components of biomaterials, e.g. functional wound dressings, to rebalance excessive angiogenesis associated with VEGF_165_/VEGFR-2 signaling, found in conditions like rheumatoid arthritis or diabetic retinopathy^69,70^.

This study revealed that GAG derivatives compete with VEGFR-2 leading to a reduced biological activity of angiogenic growth factors and, therefore, to an anti-angiogenic effect. In line with this, soluble Hep mimetics like PI-88^71–73^ and glycol-split Heps^74^ sequester angiogenic growth factors like VEGF_165_ thereby competing with endogenous cell surface HS and preventing ternary complex formation on the cell surface and receptor signaling. sGAG derivatives might exert anti-angiogenic effects through both, competition with cell-associated HS as well as growth factor receptors and, thus, be a promising option in therapeutic strategies aiming to inhibit angiogenesis.

In addition to the influence of GAG on growth factor activity, effects independent from these mediators were observed. SPR binding studies revealed an interaction of sGAG with VEGFR-2 (Fig. 5c). Xu *et al*. also found an *in situ* interaction of HS with VEGFR-2 using a proximity ligation assay^75^. Furthermore, Hep binding to VEGFR-2 was demonstrated *in vitro* in several studies^14,18,22^, while direct interaction was not observed in others^64^. Our theoretical models suggested that GAG binding to VEGFR-2 would preclude VEGF recognition.

In our SPR experiments, the binding strength of GAG toward VEGFR-2 was considerably lower compared to the binding of VEGF_165_. Such weak binding strength between GAG and VEGFR-2 did not lead to receptor activation, as the VEGFR-2 phosphorylation level in the presence of sGAG was comparable to the control without GAG and stimulating factors (Fig. 5). Interestingly, sGAG alone (Hep, sHA3, sCS3) were found to induce EC sprouting. This is in line with an enhanced EC proliferation in 3D GAG-containing hydrogels^25^. However, only low-sulfated GAGs that were crosslinked within the gel were included in these experiments, while the present study further revealed a positive effect of solute, high-sulfated GAG derivatives on ECs.

Therefore, Hep and both high-sulfated GAG derivatives might exert opposing effects on HUVECs. On one hand, they distinctly inhibited receptor binding of VEGF_165_ by interaction with this growth factor. On the other hand, they had a positive effect on HUVEC sprouting, independently from VEGF_165_. In case of Hep, there is probably a balance between these two effects, leading to no detectable effects on the biological activity of VEGF_165_ in the EC sprouting assay. This is further supported by the weaker interaction of Hep with the growth factors compared to sCS3 and sHA3. Therefore, only a portion of GAG is bound to the growth factor, while the rest might remain free and is able to activate EC sprouting. In contrast, sCS3 and sHA3, which strongly bind to VEGF_165_, remain predominantly growth factor-bound and, therefore, exerting a negative effect on VEGF_165_-mediated EC sprouting due to interference with growth factor receptor binding.

To understand the underlying mechanisms of the growth factor-independent effects of GAG, receptor-specific Abs were used to prevent GAG binding. Even though VEGFR-2 was blocked by the Ab, the added sGAG exerted a pro-angiogenic effect suggesting that VEGFR-2 is not involved. This is in line with the results on VEGFR-2 phosphorylation in the presence of sGAG alone (Fig. 5) and also with the low binding strength in SPR studies. We suggest that GAG modulate cell functions at several levels by upregulating proteins associated for instance with cell adhesion, cell signaling, matrix remodeling and endocytosis as reported for human mesenchymal stromal cells (hMSCs)^76^, which might be due to GAG internalization as shown previously^77^. In summary, sGAG do not only exert anti-angiogenic effects regarding the biological activity of VEGF_165_, but they are also pro-angiogenic in the absence of the exogenous growth factor. For the chemically high-sulfated GAG derivatives the effects were more pronounced compared to the native CS and Hep.

In addition, GAG derivatives have been shown to block inhibitors of angiogenesis, like TIMP-3^30^, indicating an additional pro-angiogenic effect. These pro- and anti-angiogenic effects could be translated into GAG-containing biomaterials to improve the healing process by tuning their composition and selected GAG type. In case of low-sulfated GAG, we expect predominantly pro-angiogenic effects on EC behavior due to the rather weak GAG-growth factor interaction leaving a considerable amount of GAG available to interact with the cells directly. This is supported by previous findings with sHA1-containing HA/collagen hydrogels^25^. For high-sulfated GAG, the strong scavenging effect on angiogenic growth factors might dominate the EC-stimulating effects.

## Conclusion

The aim of the present study was to elucidate the structure-function relationship of defined sGAG derivatives in their interplay with the angiogenic growth factor VEGF_165_ and to reveal the molecular mechanisms of the dual activity of GAG on angiogenic processes. The results obtained show that the interaction strength depends on GAG concentration, DS as well as sulfation pattern. In particular, sulfation at position C6 of GlcNAc plays an important role for these interactions. The established theoretical models in conjunction with experimental SPR and ITC results add novel insights into the mechanisms of GAG recognition by VEGF_165_ and its implication for receptor binding and, therefore, biological function. The carbohydrate backbone of the GAG had an additional influence on the interaction with VEGF_165_. GAG derivatives were found to exert growth factor-dependent but also independent effects. On one hand, GAG, upon binding to VEGF_165_, impair the interaction of the growth factor with its cognate receptor, thereby preventing downstream signaling. On the other hand, GAG alone exert pro-angiogenic effects on EC sprouting. Overall, the results obtained in this multidisciplinary approach contribute to a better understanding of the modulatory effects of GAG derivatives on angiogenic processes, which might be crucial to foster the rational design of functional biomaterials including GAG derivatives that specifically modulate angiogenesis and thereby healing processes.

## Supplementary information

Supplementary Information
