# Supplementary material for: Dual Action of Sulfated Hyaluronan on Angiogenic Processes in Relation to Vascular Endothelial Growth Factor-A
Source: Sci Rep. 2019 Dec 2;9:18143. doi: 10.1038/s41598-019-54211-0 (PMC6889296; doi:10.1038/s41598-019-54211-0)
Supplement: Supplementary file 1 — Supplementary Information [file 41598_2019_54211_MOESM1_ESM.pdf]

Supplementary Information

Scientific Reports

Dual Action of Sulfated Hyaluronan on Angiogenic Processes in Relation to Vascular Endothelial Growth Factor-A

Linda Koehler[1#], Gloria Ruiz-Gómez[2#], Kanagasabai Balamurugan[2], Sandra Rother[1], Joanna Freyse[3], Stephanie Möller[4], Matthias Schnabelrauch[4], Sebastian Köhling[3], Snezana Djordjevic[5], Dieter Scharnweber[1], Jörg Rademann[3], M. Teresa Pisabarro[2], Vera Hintze[1*]*

[1] Institute of Materials Science, Max Bergmann Center of Biomaterials, TU Dresden, Budapester Straße 27, 01069 Dresden, Germany

[2] Structural Bioinformatics, BIOTEC TU Dresden, Tatzberg 47-51, 01307 Dresden, Germany

[3] Institute of Pharmacy, Freie Universität Berlin, Königin-Luise-Straße 2+4, 14195 Berlin, Germany

[4] Biomaterials Department, INNOVENT e.V., Prüssingstraße 27 B, 07745 Jena, Germany

[5] Institute of Structural and Molecular Biology, University College London, Gower Street, Darwin Building, WC1E 6BT London, United Kingdom

# Equal contribution
* Corresponding authors

E-mail address: Maria_Teresa.Pisabarro@tu-dresden.de, +49 351 463 40071 (M. T. Pisabarro)

E-mail address: Vera.Hintze@tu-dresden.de, Tel.: +49 351 463 39389 (V. Hintze)

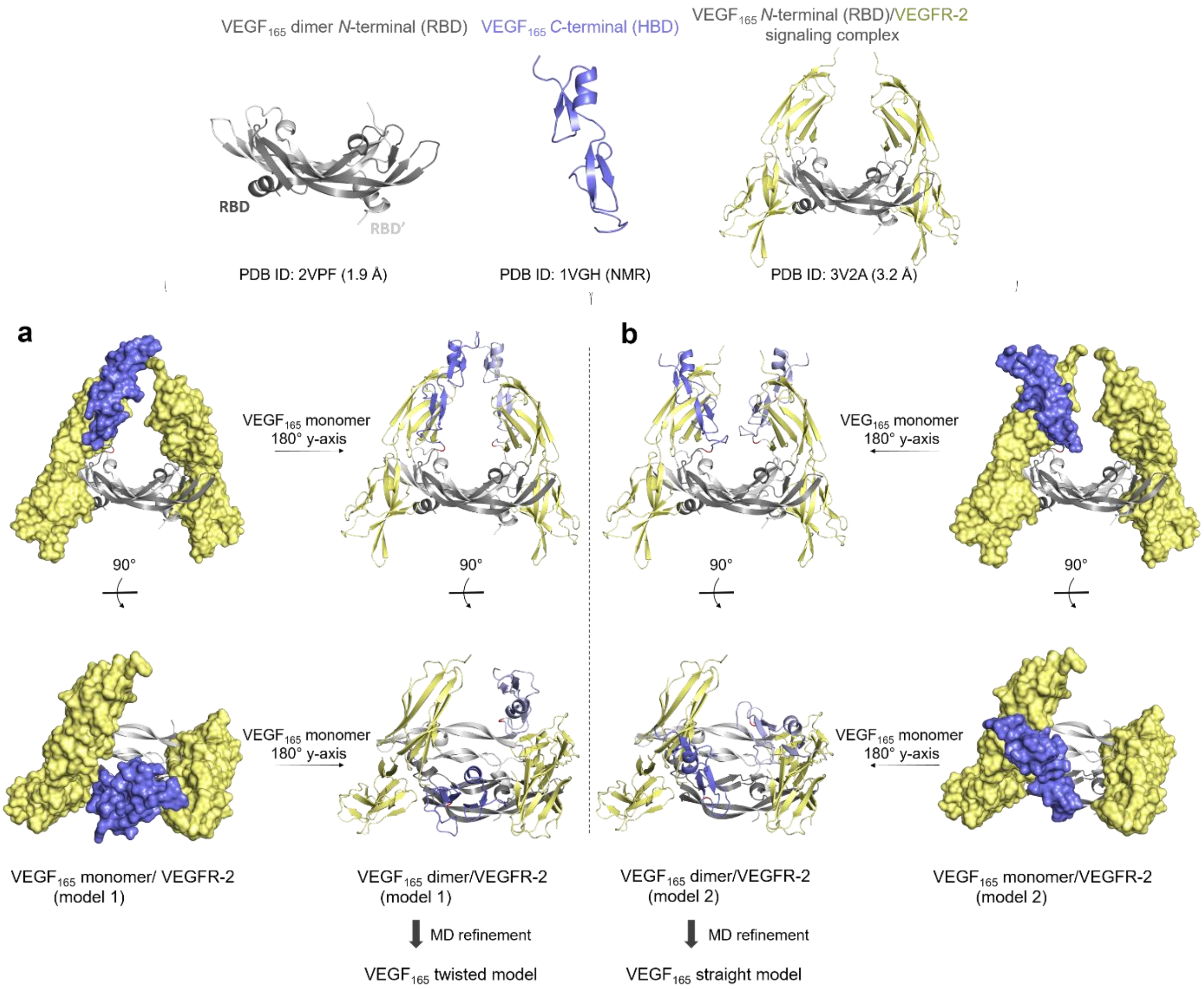

Fig. S1 Protocol for the Modelling of $VEGF_{165}$ dimer. Top: available experimental structures of *N*-terminal of $VEGF_{165}$ (receptor binding domain (RBD)), *C*-terminal (heparin binding domain (HBD)) and $VEGF_{165}$ dimer/VEGFR-2 signaling complex. Bottom: $VEGF_{165}$ models built in complex with VEGFR-2. $VEGF_{165}$ monomer model 1 (a) and model 2 (b). $VEGF_{165}$-RBD are shown in dark and light grey cartoon for each monomer, the modelled Arg110 connecting RBD and HBD is highlighted in red, $HBD_{111\text{-}114}$ and $HBD_{115\text{-}165}$ are in blue cartoon and surface, respectively, and VEGFR-2 in yellow surface and cartoon

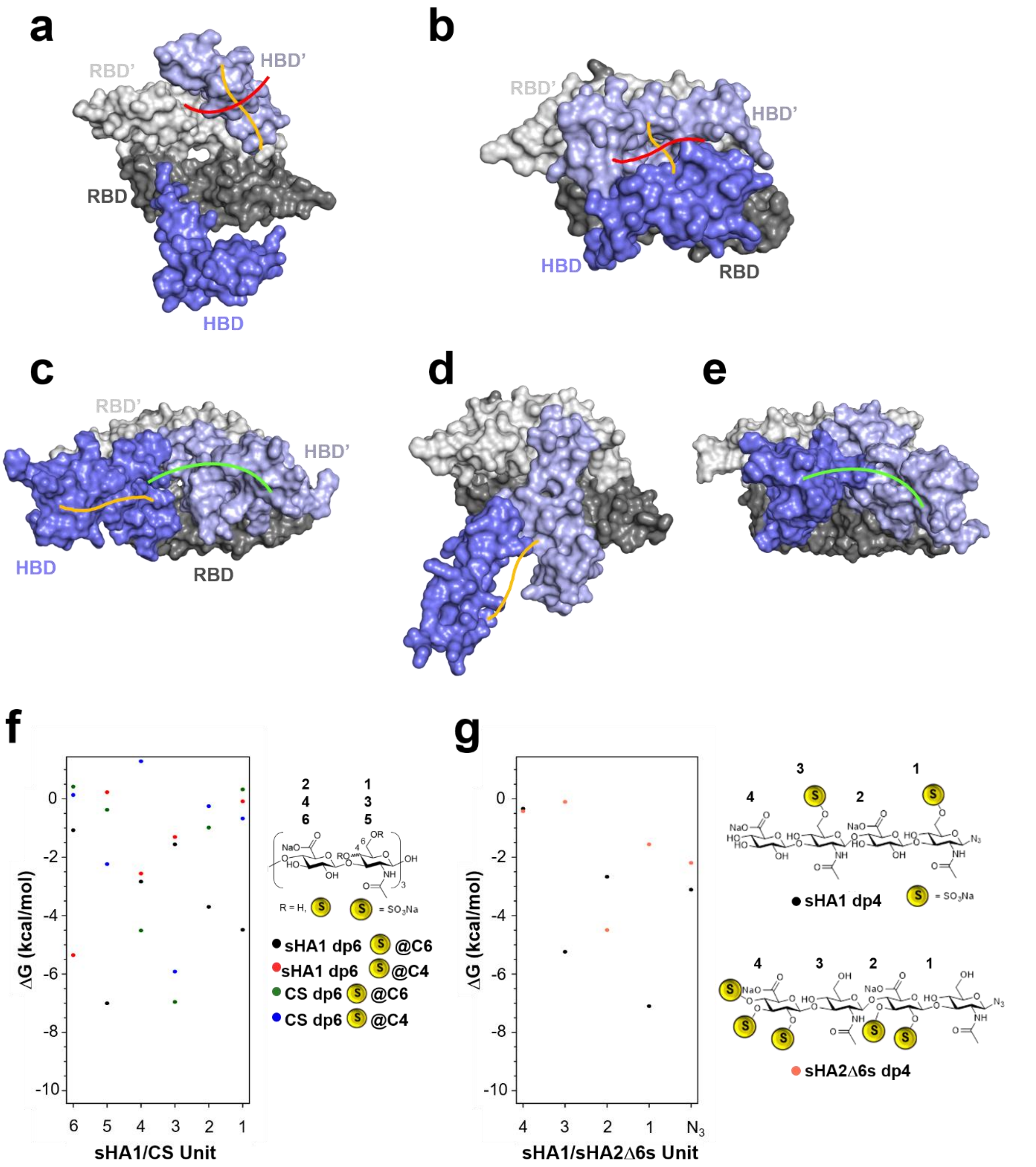

Fig. S2 Recognition of GAGs by the VEGF$_{165}$ dimer. Schematic visualization of different binding modes predicted for VEGF$_{165}$ in *twisted* conformation: parallel to HBD (orange) and perpendicular (red) from (a) docking and (b) MD-refined structure; and for the VEGF$_{165}$ in *straight* conformation: parallel (orange) and parallel-curved joining two HBDs (green) from (c) docking and (d)-(e) MD-refined structure. VEGF$_{165}$ is depicted in surface representation. RBD is highlighted in grey (dark and light representing each monomer), and HBD is highlighted in blue (dark and light representing each monomer). Per-residue binding energy contribution calculated with MM-GBSA from MD simulations of polymeric sulfated GAGs dp6 (f) and oligohyaluronans dp4 (g) in complex with VEGF$_{165}$ *twisted* conformation in the HBD-GAG-HBD stacking refined structure

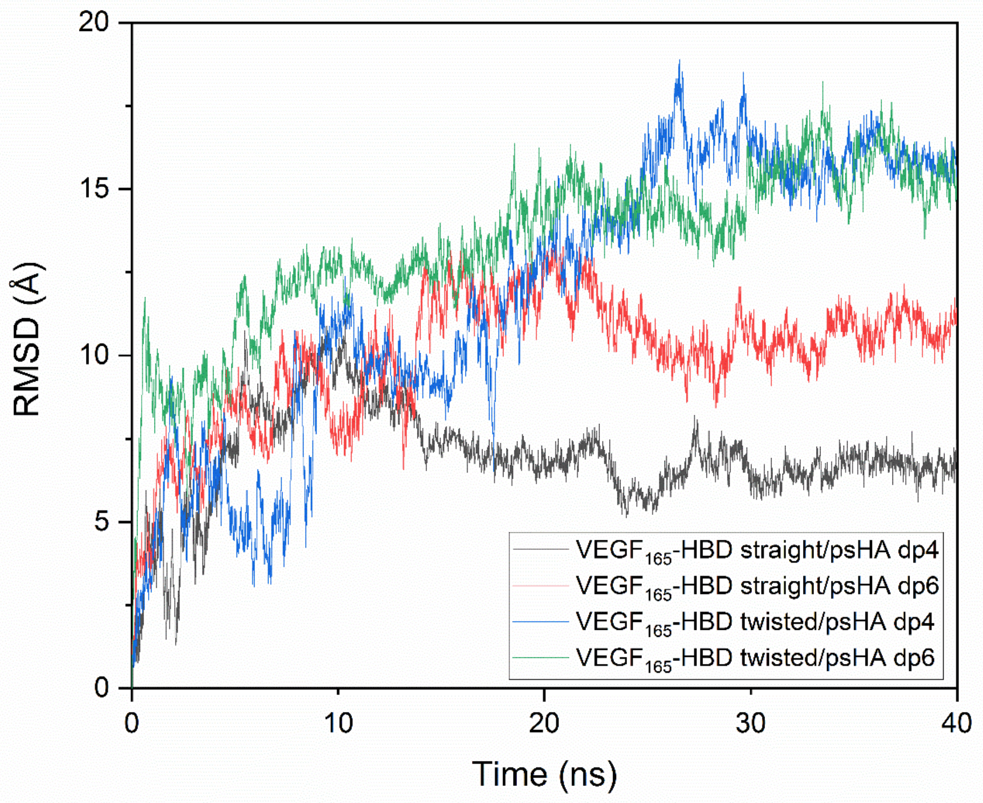

Fig. S3 RMSD for the psHA dp4 and dp6 interaction with the two $VEGF_{165}$-HBD of the $VEGF_{165}$ dimer in *twisted* and *straight* conformations for 40 ns MD simulations

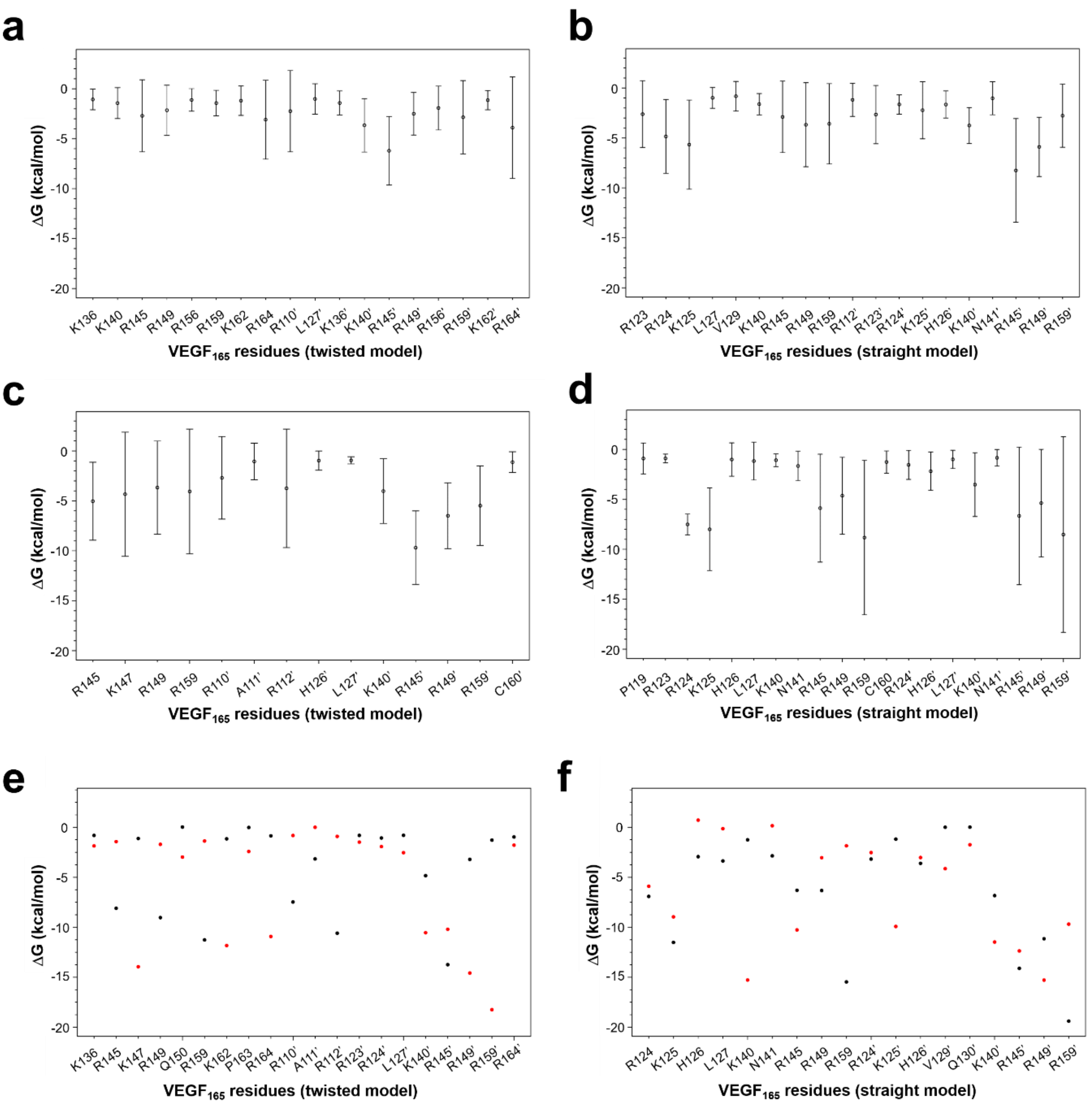

Fig. S4 Per-residue binding energy contribution calculated with MM-GBSA from MD simulations of VEGF$_{165}$ *twisted* and *straight* conformations in complex with polymeric sulfated GAGs dp6 (sHA1, CS, sHA3 and sCS3) (a)-(b), with tetrameric sulfated oligohyaluronans (sHA1, sHA2Δ6 and psHA) (c)-(d) and psHA dp4 (black dots) *vs.* psHA dp6 (red dots) (e)-(f) in the HBD-GAG-HBD stacking structure. VEGF$_{165}$ residues from the second monomer are distinguished by a dash. The errors bars represent the standard error of the mean

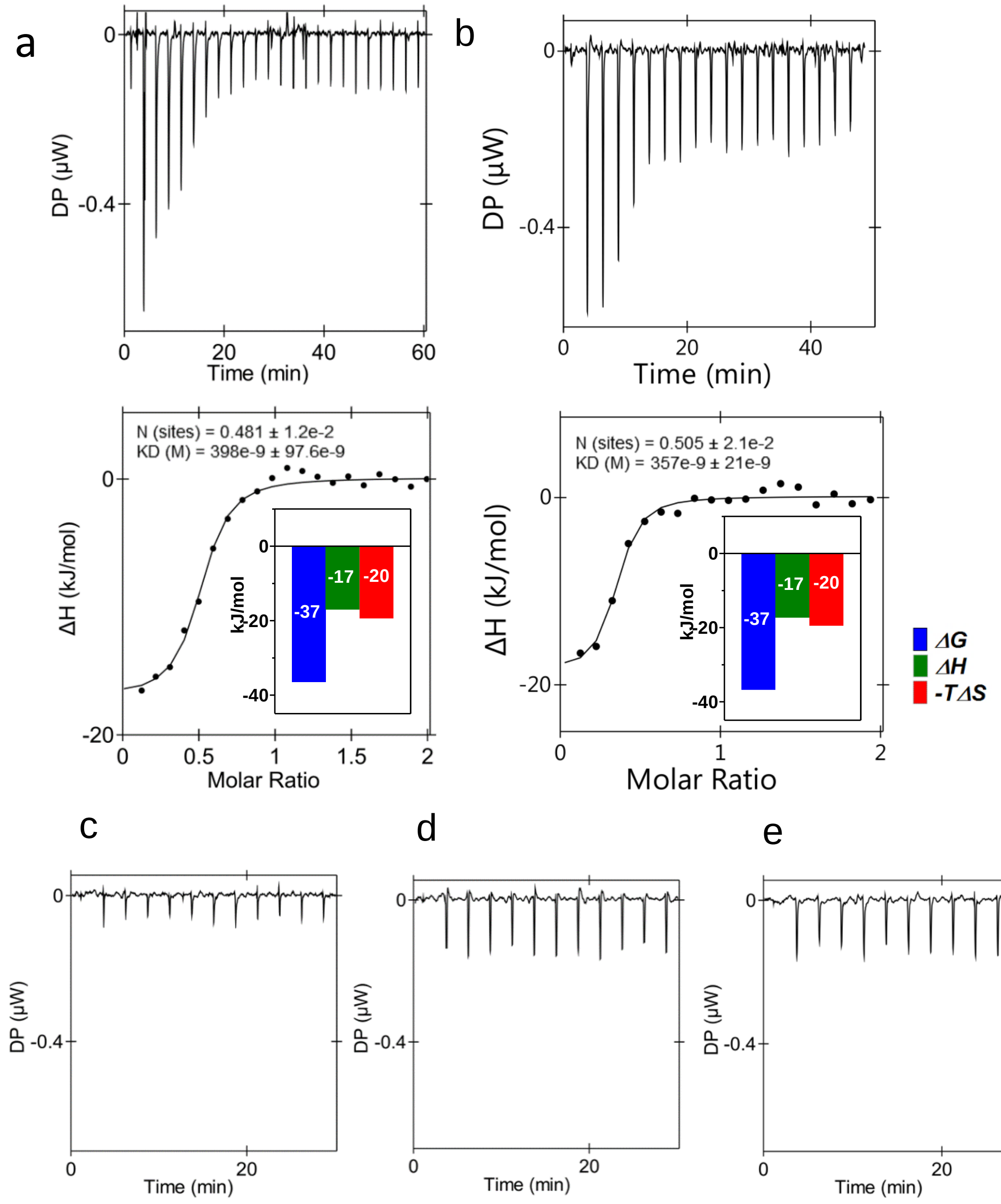

Fig. S5 Isothermal titration calorimetry data of a 60 µM solution of psHA dp4 titrated to VEGF-HBD (15 µM) is shown in (a) and (b), (c) buffer to buffer titration, (d) a 60 µM solution of psHA dp4 titrated to buffer, (e) buffer titrated to a 15 µM VEGF-HBD solution (n = 2)

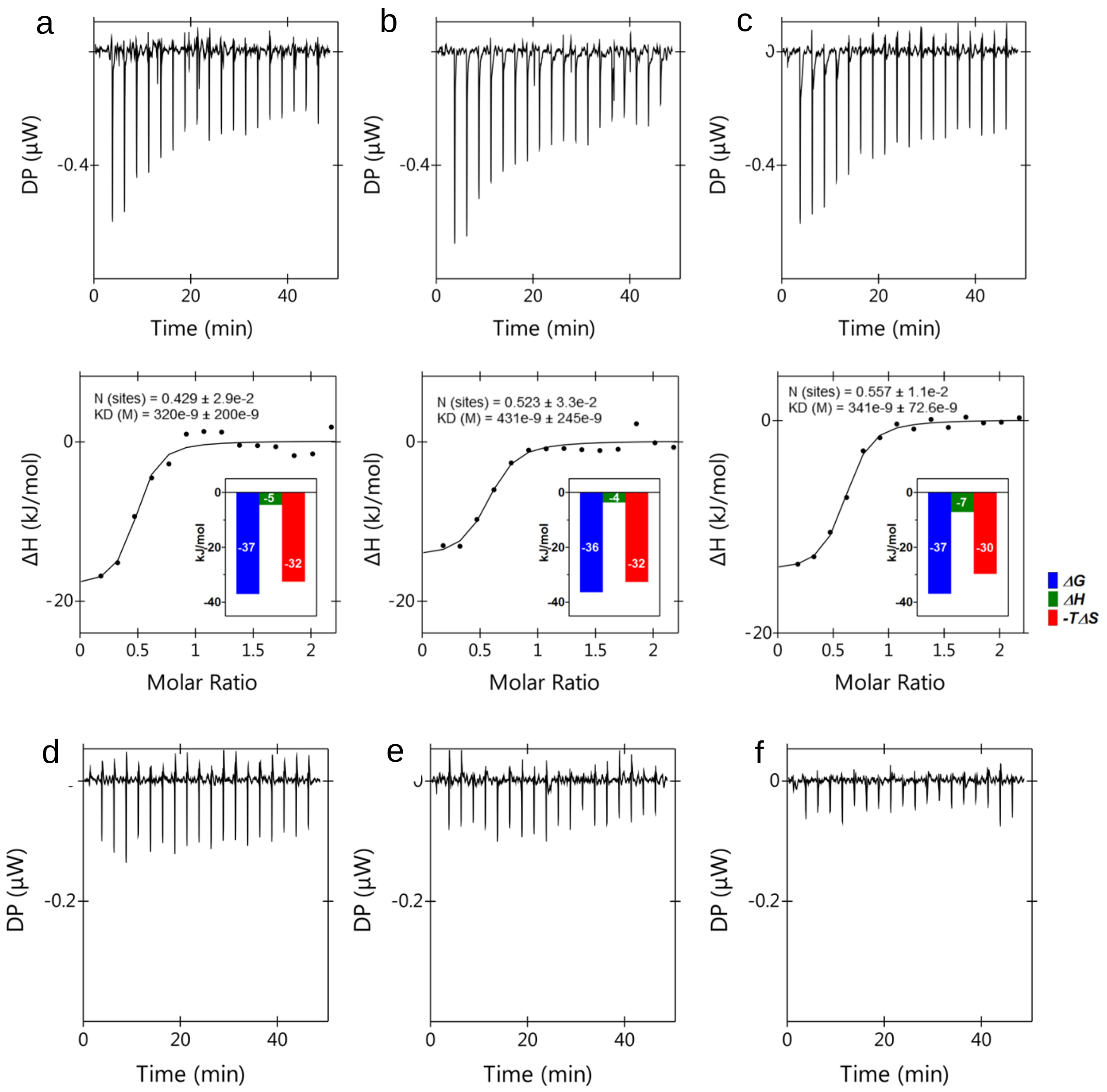

Fig. S6 Isothermal titration calorimetry data of a 60 µM solution of psHA dp4 titrated to VEGF-HBD (15 µM) is shown in (a), (b) and (c), (d) buffer to buffer titration, (e) a 60 µM solution of psHA dp4 titrated to buffer, (f) buffer titrated to a 15 µM VEGF-HBD solution (*n* = 3)

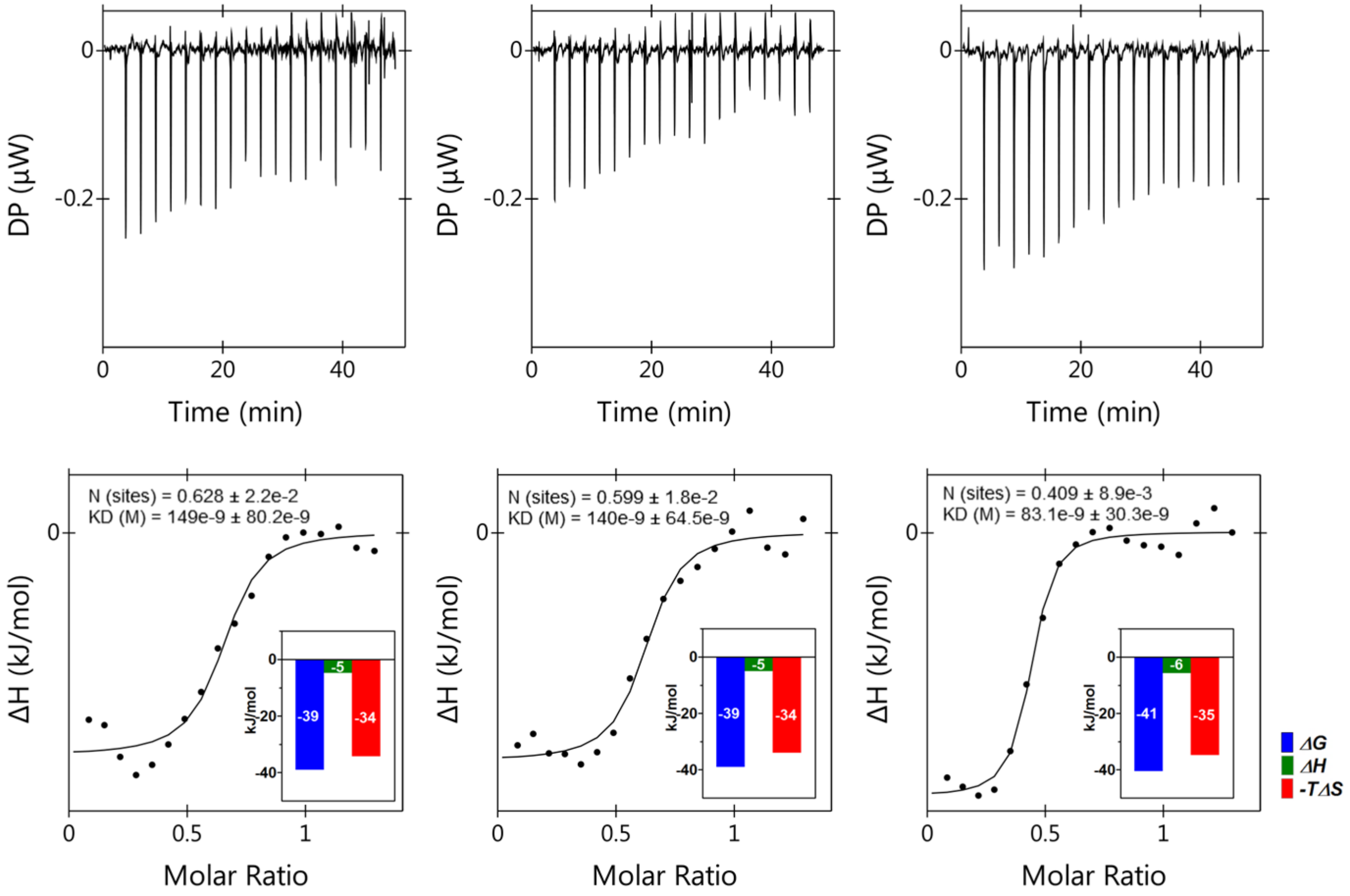

Fig. S7 Isothermal titration calorimetry data of a 50 μM solution of psHA dp6 titrated to VEGF-HBD (15 μM) is shown in (a), (b) and (c), (*n* = 3)

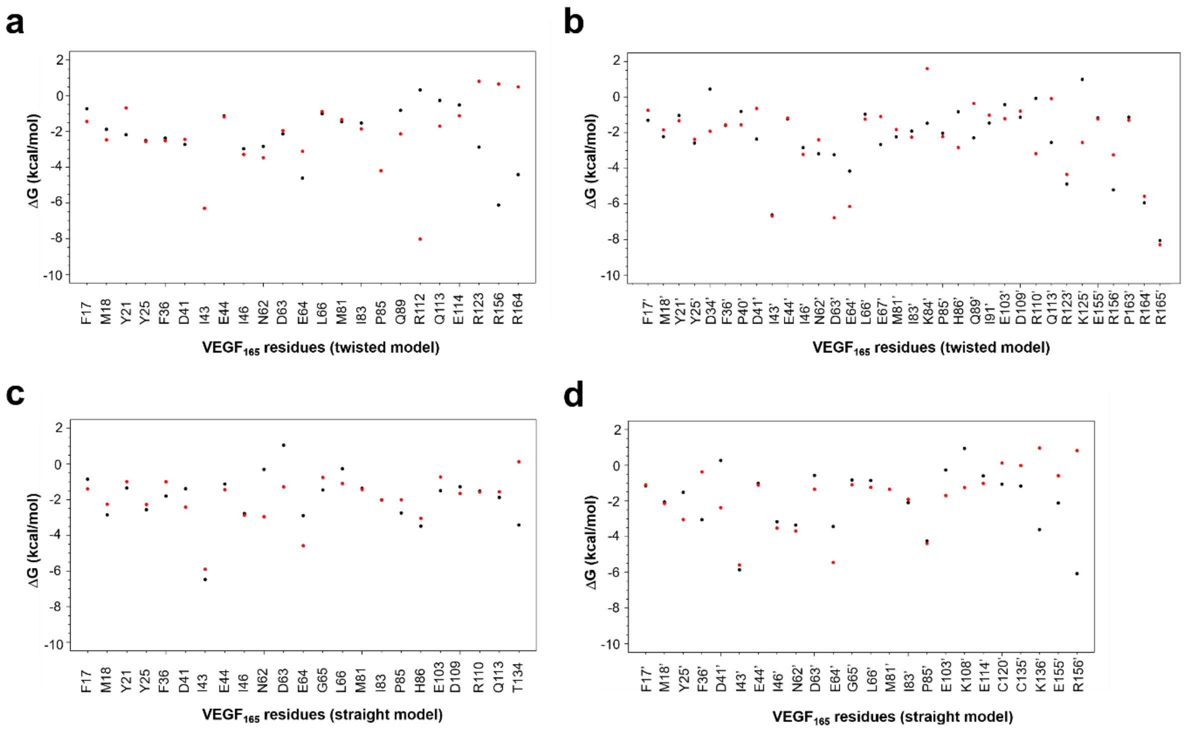

Fig. S8 Per-residue binding energy contribution calculated with MM-GBSA from MD simulations of $VEGF_{165}$ *twisted* (a) and (b), and *straight* (c) and (d) conformations in complex with two molecules of VEGFR-2 in the presence (red dots) and absence (black dots) of sHA3 dp6 . $VEGF_{165}$ residues from the second monomer in (b) and (d) are distinguished by a dash

Table S1. Schematic visualization of different binding sites of GAG derivatives in complex with VEGFR-2 (yellow cartoon) predicted by molecular docking and MM-GBSA binding free energies (left column) and interacting residues determined by per-residue energy decomposition from MD simulations. For illustrative purposes, $VEGF_{165}$-RBD (not taken into account for calculations) is shown in grey transparent cartoon (left column)

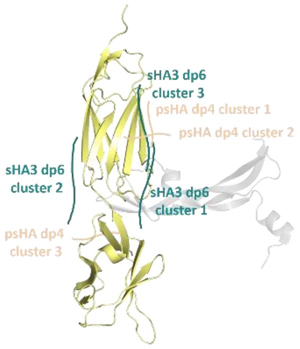

GAG	VEGFR-2 recognition site	$\Delta G_{GAG\text{-}VEGFR\text{-}2}$ (kcal/mol)
sHA3 dp6 sulfated at C4,C6,C3' (cluster 1)	K142, R249, R275, K278, T279, Q280, S281, S283, K286, K287	-22.8 ± 3.2
sHA3 dp6 sulfated at C4,C6,C3' (cluster 2)	K143, R176, R222	-9.9 ± 2.1
sHA3 dp6 sulfated at C4,C6,C3' (cluster 3)	N245, R275, L277, K278, T279, Q280, K286, K287, L289	-19.0 ± 7.1
psHA dp4 (cluster 1)	K271, R275, K278, K286	-2.0 ± 5.9
psHA dp4 (cluster 2)	K271, R275, L277, K278, T279, S281, K286, K287, L289	-43.0 ± 4.4
psHA dp4 (cluster 3)	Q132	-6.5 ± 1.3
